# Transcriptional factor six2 promotes the competitive endogenous RNA network between CYP4Z1 and pseudogene CYP4Z2P responsible for maintaining the stemness of breast cancer cells

**DOI:** 10.1186/s13045-019-0697-6

**Published:** 2019-03-04

**Authors:** Lufeng Zheng, Qianqian Guo, Chenxi Xiang, Shijia Liu, Yuzhang Jiang, Lanlan Gao, Haiwei Ni, Ting Wang, Qiong Zhao, Hai Liu, Yingying Xing, Yaohui Wang, Xiaoman Li, Tao Xi

**Affiliations:** 1 Jiangsu Key Laboratory of Carcinogenesis and Intervention, School of Life Science and Technology, China Pharmaceutical University, 24 Tong Jia Xiang, Nanjing, 210009 China; 2 Jiangsu Key Laboratory for Pharmacology and Safety Evaluation of Chinese Materia Medica, School of Pharmacy, Nanjing University of Chinese Medicine, Nanjing, 210023 China; 3 Department of Pathology, The Affiliated Hospital of Xuzhou Medical University, Xuzhou, 221002 Jiangsu China; 4 Department of Pharmacy, Jiangsu Province Hospital of TCM, Nanjing, 210023 China; 5 Department of Clinical Laboratory, Huai An First People’s Hospital, Huai An, 223300 China; 6 Department of Pathology, Jiangsu Province Hospital of TCM, Nanjing, 210023 China

**Keywords:** CYP4Z1, Pseudogene CYP4Z2P, ceRNET_CC, Six2, Stemness, Chemoresistance, Breast cancer

## Abstract

**Background:**

The expression of CYP4Z1 and the pseudogene CYP4Z2P has been shown to be specifically increased in breast cancer by our group and others. Additionally, we previously revealed the roles of the competitive endogenous RNA (ceRNA) network mediated by these genes (ceRNET_CC) in breast cancer angiogenesis, apoptosis, and tamoxifen resistance. However, the roles of ceRNET_CC in regulating the stemness of breast cancer cells and the mechanisms through which ceRNET_CC is regulated remain unclear.

**Methods:**

Transcriptional factor six2, CYP4Z1-3′UTR, and CYP4Z2P-3′UTR were stably overexpressed or knocked down in breast cancer cells via lentivirus infection. ChIP-sequencing and RNA-sequencing analysis were performed to reveal the mechanism through which ceRNET_CC is regulated and the transcriptome change mediated by ceRNET_CC. Clinical samples were used to validate the correlation between six2 and ceRNET_CC. Finally, the effects of the six2/ceRNET_CC axis on the stemness of breast cancer cells and chemotherapy sensitivity were evaluated by in vitro and in vivo experiments.

**Results:**

We revealed that ceRNET_CC promoted the stemness of breast cancer cells. Mechanistically, six2 activated ceRNET_CC by directly binding to their promoters, thus activating the downstream PI3K/Akt and ERK1/2 pathways. Finally, we demonstrated that the six2/ceRNET_CC axis was involved in chemoresistance.

**Conclusions:**

Our results uncover the mechanism through which ceRNET_CC is regulated, identify novel roles for the six2/ceRNET_CC axis in regulating the stemness of breast cancer cells, and propose the possibility of targeting the six2/ceRNET_CC axis to inhibit breast cancer stem cell (CSC) traits.

**Electronic supplementary material:**

The online version of this article (10.1186/s13045-019-0697-6) contains supplementary material, which is available to authorized users.

## Introduction

Human tumors are composed of heterogeneous cells, in which cancer stem cells (CSCs), here defined as the tumor cells specifically endowed with self-renewal and tumor-seeding potential, have been regarded as the main drivers of tumor progression and chemoresistance [1]; however, the mechanisms underlying CSC maintenance are not well defined, and currently, no drugs directly kill CSCs. In fact, we have only a scattered understanding of the cellular mechanisms that contribute to CSC attributes. Notably, because tumorigenesis and chemoresistance are led by the deregulation of gene networks, single gene expression analysis cannot completely explain chemotherapy or tumor recurrence, which should be assessed by several synergistic factors.

Competitive endogenous RNAs (ceRNAs) are defined as the transcripts that share common miRNAs and can coregulate each other’s expression [2]. ceRNAs play critical roles in tumor progression; for example, the long non-coding RNA UICLM can facilitate colorectal cancer metastasis by acting as a ceRNA for miR-215 [3] and ROR promotes pancreatic cancer progression by acting as a ceRNA for Nanog [4]. We and others have shown that the expression of CYP4Z1 and the pseudogene CYP4Z2P are significantly and specifically higher in breast cancer tumor tissues [5–7]. Furthermore, we revealed that CYP4Z1 and the pseudogene CYP4Z2P form a ceRNA network via competiting with several miRNAs, such as miR-211, miR-125a-3p, miR-197, miR-1226, and miR-204, here called ceRNET_CC, and both we and others have shown that these shared miRNAs exert tumor suppressive effects [8–12]. We further demonstrate that ceRNET_CC promotes breast cancer angiogenesis [6] and tamoxifen resistance [5] and suppresses breast cancer apoptosis [13]; these effects suggest that suppression of ceRNET_CC may allow for inhibition of breast cancer. Due to the roles of ceRNET_CC in promoting breast cancer, here, we focused on understanding the mechanisms underlying the progression of ceRNET_CC and the roles of ceRNET_CC in regulating the stemness of breast cancer. Transcript overexpression occurs through transcriptional and epigenetic control mechanisms in the vast majority of cancers, as demonstrated by Rinath et al., who uncovered a role for the RUNX2-ER (runt-related transcription factor 2-estrogen receptor) complex in stimulating the transcription of a set of genes, including most notably the stem cell factor Sox9, which promotes proliferation and a metastatic phenotype [14], and TRIM28, which interacts with EZH2 and SWI/SNF to activate genes that promote mammosphere formation [15]. A bioinformatics method was then used to predict the transcriptional factors binding to the promoters of CYP4Z1 and the pseudogene CYP4Z2P, and transcriptional factor six2 attracted our attention based on its critical roles in organ development and promoting roles in cancers [16–18].

Transcriptional factor six2 activity is required to maintain the mesenchymal progenitor population in an undifferentiated state [16], and cells autonomously regulate a multipotent nephron progenitor population throughout mammalian kidney development [17]. Additionally, haploinsufficiency for the six2 gene increases nephron progenitor proliferation, promoting branching and nephron number [19]. These processes are normally maintained by stem cells, and the expression of genes involved in embryonic development is usually reinstated in tumors. Additionally, previous studies have revealed that several cell fate regulators (DeltaNp63, Slug, Sox9, and miR-200c) are molecular links between mammary stem cells and breast tumor-initiating cells that drive renewal activity in both normal and cancerous mammary gland tissues [20–22]. Based on these data, we speculated that six2 could drive CSC progression. Previous studies have shown that breast cancer patients with higher six2 levels have shorter time to both relapse and metastasis [18, 23]. Additionally, increased expression and decreased methylation of six2 are correlated with increased tumor size, clinical stage, vascular invasion, and unfavorable histological differentiation in Wilms’ tumor [24]. Moreover, a systematic meta-analysis revealed that higher six2 expression is associated with a greater possibility of tumorigenesis and predicted poor overall survival (OS) in non-small-cell lung cancer (NSCLC) and poor relapse-free survival (RFS) in lung adenocarcinoma (ADC) [25]. However, the molecular mechanisms underlying six2-mediated oncogenic effects, and the downstream effectors of six2 are not fully understood.

In the present study, we showed that the expression of six2, CYP4Z1, and pseudogene CYP4Z2P was significantly increased in breast cancer tumors and that six2 could directly bind to the 5′-TCAG-3′ motif in the promoter of CYP4Z1 and CYP4Z2P and thus promote the progression of ceRNET_CC. Notably, this novel six2/ceRNET_CC regulatory axis was responsible for the stemness and chemoresistance of breast cancer cells. Importantly, the expression of six2, CYP4Z1, and the pseudogene CYP4Z2P was negatively correlated with the OS of breast cancer patients, and the expression of these genes was positively correlated with one another, underscoring the critical roles of this regulatory axis in breast cancer progression. A broader understanding of six2-dependent regulation on ceRNET_CC is needed to effectively target therapy-resistant breast cancer cells with stemness characteristics and address the challenges of tumor heterogeneity.

## Materials and methods

### Clinical samples

Paraffin-embedded breast cancer tissue samples were obtained in our previous work [26]. Eight pairs of fresh breast cancer and normal adjacent tissues were collected from Huai An First People’s Hospital and Jiangsu Province Hospital of TCM between September 2017 and January 2018. Written informed consent from all patients and approval of the hospital ethics review committees were obtained. Online clinical posited data (http://www.firebrowse.org/), Breast Cancer Gene-Expression Miner (version 4.0; http://bcgenex.centregauducheau.fr/), and R2: Genomics Analysis and Visualization Platform (http://hgserver1.amc.nl/cgi-bin/r2/main.cgi) were used for the analyses of gene expression and correlation. The diagnostic values of the genes were analyzed by KM plotter (http://kmplot.com) to obtain KM survival plots in which the number at risk is indicated below the main plot [27]. Hazard ratio, 95% confidence intervals, and log-rank *P* values were calculated and displayed on the webpage.

### Cell culture and chemical reagents

The human breast cancer cell lines MCF-7, MDA-MB-231, and HEK293T were preserved in our laboratory. Adriamycin-resistant MCF-7-Adr cells were purchased from KeyGen BioTECH (Nanjing, China). The cell line was authenticated every year through short tandem repeat (STR) DNA profiling. HEK293T and MCF-7 cells were cultured in DMEM (Gibco, Grand Island, NY, USA), MCF-7-Adr cells were cultured in 1640 medium (Gibco), and MDA-MB-231 cells were cultured in L-15 medium (Gibco) at 37 °C under a humidified atmosphere with 5% CO_2_. All of the media were supplemented with 10% FBS (Gibco), 80 U/ml penicillin, and 0.08 mg/ml streptomycin. PI3K inhibitor (LY-294002) and ERK1/2 inhibitor (VX-11e) were purchased from APExBIO. Adriamycin was purchased from Zhongda Hospital Southeast University.

### Quantitative real-time PCR (qRT-PCR)

Total RNA from the cells was extracted using TransZol Up (Cat. No. ET111-01, TransGen Biotech, Beijing, China) following the manufacturer’s recommendation. Total RNA from paraffin-embedded breast cancer tissues was extracted using a total RNA extraction kit for paraffin-embedded tissues (Cat. No. DP439, TianGen Biotech, Beijing, China) according to standard protocols. Then, complementary DNA (cDNA) was reverse-transcribed using M-MLV (H-) Reverse Transcriptase (Cat. No. R021-01, Vazyme, Nanjing, China) according to the manufacturer’s protocol. qRT-PCR was performed with AceQ Universal SYBR qPCR Master Mix (Cat. No. Q511-02, Vazyme). A melting curve analysis was performed routinely to check the amplification specificity. cDNA templates were analyzed in triplicate, and GAPDH was used as an internal control. The relative expression level of each transcript was calculated by the 2^-△△ct^ method. The qRT-PCR primers are described in Additional file 1: Table S1.

### Western blotting

The detailed procedure was described in our previous study [26]. Protein in fresh tissues was extracted using total protein extraction kit (Invent, USA) following the manufacturer’s recommendation. β-actin or GAPDH was used as an internal reference. Detailed information on the antibodies used in this work is given in Additional file 2: Table S2.

### Fluorescence-activated cell sorting

CD24 and CD44 expression was analyzed in cells derived from monolayer cultures following dissociation in trypsin-EDTA at 37 °C. At least 1 × 10^6^ cells were pelleted by centrifugation at 300×*g* and 4 °C for 5 min. Then, cells were washed in PBS, re-suspended with anti-CD24-PE (BD Biosciences, USA) and anti-CD44-APC (BD Biosciences, USA), and then incubated at 4 °C for 30 min in the dark. The labeled cells were washed using PBS and analyzed using a flow cytometer (BD, USA). The negative fraction was determined using appropriate isotype controls.

### Chromatin immunoprecipitation assay

A chromatin immunoprecipitation (ChIP) assay was performed using the EZ-Magna ChIP™ A/G Chromatin Immunoprecipitation Kit (Cat. No. 17–10086, Merck) following the manufacturer’s protocols. Primers flanking the six2 binding sites on the promoters of CYP4Z1 and pseudogene CYP4Z2P were used for qRT-PCR. The sequences of the primers for ChIP analysis were denoted in Additional file 3: Table S3.

### ChIP-sequencing and data assay

ChIP-sequencing analysis was performed by GENEWIZ (Suzhou, China). ChIP-seq raw reads were aligned to a human reference genome (hg19) using cutadapt (version 1.9.1) to pass filter data and acquire clean data. Up to mismatch per read was allowed. The quality of the sequencing data was assessed using FastQC (v0.10.1), and only uniquely mapped reads were kept for downstream analysis. The data are available in the Gene Expression Omnibus (GEO) database as GSE117145.

### RNA sequencing and data analysis

RNA sequencing and data analysis were conducted by Novogene (Beijing, China). The data are available in the Gene Expression Omnibus (GEO) database as GSE116984.

### Tissue microarray analysis

A tissue microarray including 30 breast cancer tissues and 30 normal adjacent tissues was purchased from OUTDO IVD (Shanghai, China). Further immunohistochemistry was performed to detect the expression of six2 following the protocols described in our recent work [28].

### Small interfering RNA transfection

When cells confluency reached 50%, cells were transfected with a final concentration of 50 nM small interfering RNA (siRNA) or 50 nM NC (si-NC, inhibitor-NC, mi-NC) which were synthesized by Biomics Biotechnology (Nantong, China) using jetPRIME (Polyplus Transfection, France) following the manufacturer’s protocols. The siRNA sequences are mentioned in Additional file 4: Table S4.

### Cell spheroid formation assay

A mammosphere formation assay was performed using MammoCult™ Human Medium Kit (STEMCELL Technologies, Canada). A total of 3 × 10^3^ cells were mixed with 500 μl of Complete MammoCult™ Medium in the presence of 4 μg/ml Heparin (STEMCELL Technologies, Canada) and 0.48 μg/ml hydrocortisone (STEMCELL Technologies, Canada) and seeded in 24-well ultra-low attachment plates (Corning, USA) for 7 days. The spheres were counted and photographed. All images were obtained with a Leica DMI microscope (DE).

### Lentivirus package, stable cell lines, and plasmid construction

To construct stable expression cells, the six2 coding area or the CYP4Z1 3′UTR or CYP4Z2P 3′UTR sequences were subcloned into pLVX-ZsGreen. siRNA oligos were purchased from GenePharma (Shanghai, China). After annealing, double-strand oligos were inserted into the lentiviral pLKO.1-puro vector (Addgene). To package lentivirus, HEK293T cells were co-transfected using Lentifectin (ABM, USA) with the lentiviral vector and packaging vectors psPAX2 and pMD2.G. MCF-7 and MDA-MB-231 cells were infected with the virus in the presence of 2 μg/ml polybrene. Cells infected with Plko.1-derived vectors were selected with puromycin (Sigma, 2 μg/ml) for 2 weeks. Cells infected with pLVX-ZsGreen-derived vectors were selected by fluorescent cell sorting. Western blot and qRT-PCR analyses were used to verify expression levels. MCF-7 cells stably overexpressing CYP4Z1 3′UTR, CYP4Z2P 3′UTR, and six2 were designated as MCF-7-Z1-UTR, MCF-7-Z2P-UTR, and MCF-7-six2, respectively. MCF-7 cells stably knocking down CYP4Z1 3′UTR, CYP4Z2P 3′UTR, and six2 stable were denoted as MCF-7-Plko-Z1, MCF-7-Plko-Z2P, and MCF-7-Plko-six2, respectively. MDA-MB-231 cells stably overexpressing six2 were designated as 231-six2, while those stably knocking down CYP4Z1 3′UTR, CYP4Z2P 3′UTR, and six2 were denoted as 231-Plko-Z1, 231-Plko-Z2P, and 231-Plko-six2, respectively. The sequences of the primers used for plasmid constructions were listed in Additional file 5: Table S5.

### Luciferase reporter assay

The promoter sequences of CYP4Z1 (1–2403 bp) and the pseudogene CYP4Z2P (1–2998 bp) were inserted into the pGL3-promoter vector, and truncations of these sequences were also inserted into the pGL3-promoter vector. Then, potential six2 binding sites were mutated using the Fast Mutagenesis Kit V2 (Vazyme, China) following the manufacturer’s instructions and inserted into the pGL3-promoter vector as well. All of the abovementioned constructs were verified by DNA sequencing before use. To analyze the activity of the abovementioned constructs, they were individually co-transfected with β-gal and a six2 overexpression vector into MCF-7 cells. Seventy-two hours later, the luciferase activity was measured by a POLARstar Omega multimode microplate reader according to the manufacturer’s protocol and normalized to β-gal. All primer sequences for this experiment are listed in Additional file 6: Table S6**.**

### Chemoresistance or sensitivity assay

Cell viability was assessed using MTT (Amresco, USA) staining. Cells were seeded at 3 × 10^3^ cells/well in 96-well cell plates overnight and then treated with different concentrations of adriamycin for 48 h. During the last 3.5 h, the cells were exposed to MTT (5 mg/ml) and the resulting formazan crystals were dissolved in 150 μl of DMSO and measured using a spectrophotometer(BIO-RAD) at a test wavelength of 490 nM. The experiments were conducted in triplicate.

### In vivo tumorigenesis

The procedure was referenced in our previous work [28]. Briefly, six-week-old male athymic BALB/c nude mice were purchased from the Model Animal Research Center of Nanjing University, housed and fed under standard pathogen-free conditions. For the tumor-limiting dilution assay, 1 × 10^6^ and 1 × 10^5^ MCF-7 cells, 1 × 10^5^ and 1 × 10^4^ MDA-MB-231 cells, or 1 × 10^5^ MCF-Adr cells receiving different treatment were orthotopically implanted in the inguinal mammary gland of mice. On day 8, all the mice were euthanized and tumor tissues were collected and weighed. The stem cell frequencies were calculated using an ELDA (http://bioinf.wehi.edu.au/software/elda/) [29]. All the animal experiments were carried out with the approval of the Ethics Committee for Animal Experimentation of China Pharmaceutical University.

### Statistical analysis

GraphPad Prism 5.01 software (GraphPad Software, Inc., La Jolla, CA, USA) was used for the statistical analysis. All the data were obtained from at least three independent experiments and are presented as the means ± standard deviations (SDs). Datasets with only two groups were analyzed using Student’s *t* test. Differences between multiple groups were analyzed by one-way analysis of variance with the Tukey-Kramer post hoc test. *P* < 0.05 was considered indicative of a statistically significant difference.

## Results

### CeRNET_CC promotes the stemness of breast cancer cells in vitro

**Fig. 1:**
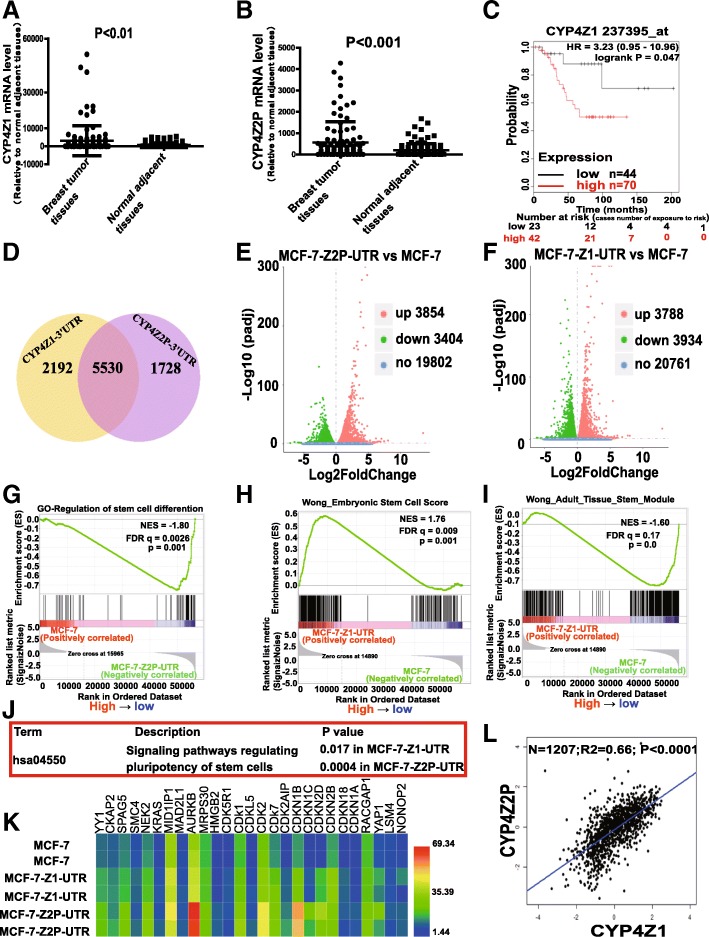
CeRNET_CC promotes the stemness of breast cancer cells in vitro*.* **a**, **b** The mRNA levels of CYP4Z1 and the pseudogene CYP4Z2P were detected in breast cancer and normal adjacent tissues via online deposited data (*n* = 1207, error bars denote the ± SDs, *P* < 0.01). **c** KM-plotter (http://kmplot.com) survival curves based on the analysis of a published microarray dataset from breast cancer patients showed the OS probability of patients separated into low and high CYP4Z1 levels. **d** Venn diagram showing overlap of genes in MCF-7-Z1-UTR and MCF-7-Z2P-UTR cells. **e**, **f** Genes with expression levels that were upregulated or downregulated in MCF-7-Z2P-UTR (**e**) or MCF-7-Z1-UTR (**f**) cells relative to those in MCF-7 cells. **g**–**i** Enrichment of a stem cell signature in a GSEA of genes regulated in MCF-7-Z2P-UTR and MCF-Z1-UTR cells. NES, normalized enrichment score; FDR, false discovery rate. **j** Functional annotation analysis of genes coordinately activated by CYP4Z1-3′UTR- or CYP4Z2P-3′UTR. **k** Heat map showing the mean expression values of genes related to epithelial cancer stem cells in MCF-7 cells with CYP4Z2P-3′UTR and CYP4Z1-3′UTR overexpression (fold change > 2 compared with MCF-7 cells, *P* < 0.05). **l** Pearson correlation analysis of the expression of CYP4Z1 and the pseudogene CYP4Z2P in breast cancer tissues (*n* = 1207, *P* < 0.0001)

The expression levels of CYP4Z1 and its pseudogene CYP4Z2P were initially examined in breast tumor and normal adjacent tissues via online clinical deposited data (http://www.firebrowse.org/). As shown in Fig. 1a, b, the expression of CYP4Z1 and the pseudogene CYP4Z2P was significantly increased in breast tumor tissues, which is consistent with our previous work in which CYP4Z1 and pseudogene CYP4Z2P expression was detected in clinical samples [26, 30]. KM-plotter analysis (http://kmplot.com) indicated that CYP4Z1 expression was negatively correlated with the OS of breast cancer patients (Fig. 1c). We defined the CYP4Z1-3′UTR- or CYP4Z2P-3′UTR-regulated transcriptome in MCF-7 cells with CYP4Z1- or CYP4Z2P-3′UTR overexpression and subsequently performed expression profiling, and the results revealed a substantial and highly statistically significant overlap between genes regulated by both CYP4Z1-3′UTR and CYP4Z2P-3′UTR in MCF-7 cells (Fig. 1d). Additionally, CYP4Z1-3′UTR and CYP4Z2P-3′UTR activated (3854 vs. 3788) or repressed (3404 vs. 3934) similar numbers of genes in MCF-7 cells (Fig. 1e, f). Further gene set enrichment analysis (GSEA) of this dataset revealed that a negative enrichment of stem cell-differentiated signatures was observed in MCF-7 cells with CYP4Z2P-3′UTR overexpression (Fig. 1g), and the top positive signatures associated with CYP4Z1-3'UTR overexpression were the embryonic stem cell function and adult tissue stem modules (Fig. 1h, i) [31], both of which are indicative of a stemness phenotype. Functional annotation analysis revealed that the overexpression of CYP4Z2P-3′UTR or CYP4Z1-3′UTR activated signaling pathways regulating the pluripotency of stem cells (Fig. 1j). Among the differentially expressed genes, we identified a set of gene signatures related to epithelial cancer stem cells in MCF-7 cells with CYP4Z2P-3′UTR and CYP4Z1-3′UTR overexpression, including YY1, KRAS, YAP1, and HMGB2 (Fig. 1k) [31]. Consistent with the activation of stem cell function, we detected an increase in a set of cell cycle-related genes, namely CDK5R1, CDK1, CDK2, and CDK7 (Fig. 1). Notably, the expression of CYP4Z1 and CYP4Z2P displayed a positive correlation in clinical breast cancer samples (*P* < 0.001, Fig. 1l).

As previous studies have demonstrated that non-adherent spheres are highly enriched for CSCs [32, 33], the expression levels of CYP4Z1 and its pseudogene CYP4Z2P were examined in non-adherent MCF-7 spheres and parental cells. qRT-PCR results showed that non-adherent MCF-7 spheres displayed significantly higher levels of CYP4Z1 and CYP4Z2P relative to monolayer cultures of MCF-7 cells (Additional file 7: Figure S1A). We then determined whether the overexpression of CYP4Z1- or CYP4Z2P-3′UTR conferred stemness upon breast cancer cells in vitro. First, MCF-7 cells with CYP4Z1- or CYP4Z2P-3′UTR stable overexpression or knockdown were subjected to a spheroid formation assay. The infection efficiency was confirmed by qRT-PCR (Additional file 7: Figure S1B and C). Both the sphere size and number were increased in MCF-7 cells with CYP4Z1- or CYP4Z2P-3′UTR overexpression, whereas the knockdown of CYP4Z1- or CYP4Z2P-3′UTR exerted the opposite effects (Additional file 7: Figure S1D). Additionally, the overexpression of CYP4Z1- or CYP4Z2P-3′UTR increased the CD44^+^/CD24^−^ population, which has been identified as having breast CSC markers [34] (Additional file 7: Figure S1E). Moreover, the expression of several pluripotent transcription factors, namely Oct3/4, ALDH1, Nanog, and Sox2, was increased in cells with CYP4Z1- or CYP4Z2P-3′UTR overexpression, whereas the knockdown of CYP4Z1- or CYP4Z2P-3′UTR yielded the opposite effects (Additional file 7: Figure S1F and S1G). These results were recapitulated in MDA-MB-231 cells with or without the knockdown of CYP4Z1- or CYP4Z2P-3′UTR. qRT-PCR analysis confirmed the knockdown efficiency of shRNAs against CYP4Z1- or CYP4Z2P-3′UTR (Additional file 8: Figure S2A and B). As expected, the knockdown of CYP4Z1- or CYP4Z2P-3′UTR resulted in fewer primary mammospheres than control cells (Additional file 8: Figure S2C) and decreased the CD44^+^/CD24^−^ population (Additional file 8: Figure S2D). Additionally, the expression of stemness markers was inhibited in MDA-MB-231 cells with CYP4Z1- or CYP4Z2P-3′UTR knockdown (Additional file 8: Figure S2E and F). Notably, we assessed the expression correlation between Nanog and CYP4Z1 or CYP4Z2P across basal-like breast cancer subtypes using Breast Cancer Gene-Expression Miner (version 4.0; http://bcgenex.centregauducheau.fr/) and found that Nanog expression is positively correlated with CYP4Z1 or CYP4Z2P expression in basal-like breast cancer subtypes (Additional file 8: Figure S2G and S2H). These results indicate that ceRNET_CC is engaged in tumor stemness in breast cancer.

### ceRNET_CC promotes the tumor-initiating potential of breast cancer cells in vivo

**Fig. 2:**
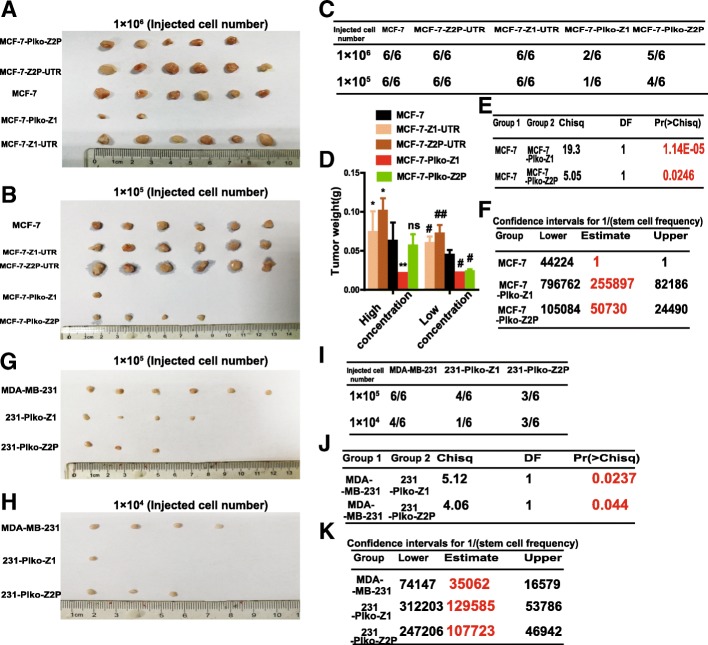
CeRNET_CC promotes the tumor-initiating potential of breast cancer cells in vivo. **a**–**c** Images (**a**, **b**) and number (**c**) of tumors harvested when serially diluted MCF-7, MCF-7-Z1-UTR, MCF-7-Z2P-UTR, MCF-7-Plko-Z1, and MCF-7-Plko-Z2P were seeded. **d** Weight of tumors harvested when serially diluted cells depicted in **a** were seeded. **e**, **f** The limiting dilution analysis was performed using ELDA software. **g**–**i** Images (**g**, **h**) and number (**i**) of tumors harvested when serially diluted MDA-MB-231, 231-Plko-Z1, and 231-Plko-Z2P were seeded. **j**, **k** Pr values, associated probabilities (**j**), and TIC (tumor-initiating cell) frequencies (**k**) of cells depicted in **g**

We further investigated whether ceRNET_CC facilitates the tumor-initiating potential of breast cancer cells in vivo. We compared the capacity of MCF-7 cells with CYP4Z1- or CYP4Z2P-3′UTR overexpression or knockdown to seed tumors at limiting dilutions. Although all of the cell lines could form tumors at a density of 1 × 10^6^ and 1 × 10^5^ cells, CYP4Z1- or CYP4Z2P-3′UTR overexpressed cells showed increased tumor size and weight (Fig. 2a–d) while knockdown of CYP4Z1- or CYP4Z2P-3′UTR decreased the tumor-initiating ability of MCF-7 cells (Fig. 2a–f). Additionally, we performed an in vivo tumorigenic assay with MDA-MB-231 cells after CYP4Z1- or CYP4Z2P-3′UTR knockdown and showed that the knockdown of CYP4Z1- or CYP4Z2P-3′UTR remarkably reduced the tumor-initiating potential of MDA-MB-231 cells (Fig. 2g–k). Therefore, our results demonstrate that ceRNET_CC could promote the stemness of breast cancer cells.

### ceRNET_CC promotes the stemness of breast cancer cells partly through the hTERT/PI3K/Akt and ERK1/2 pathways

**Fig. 3:**
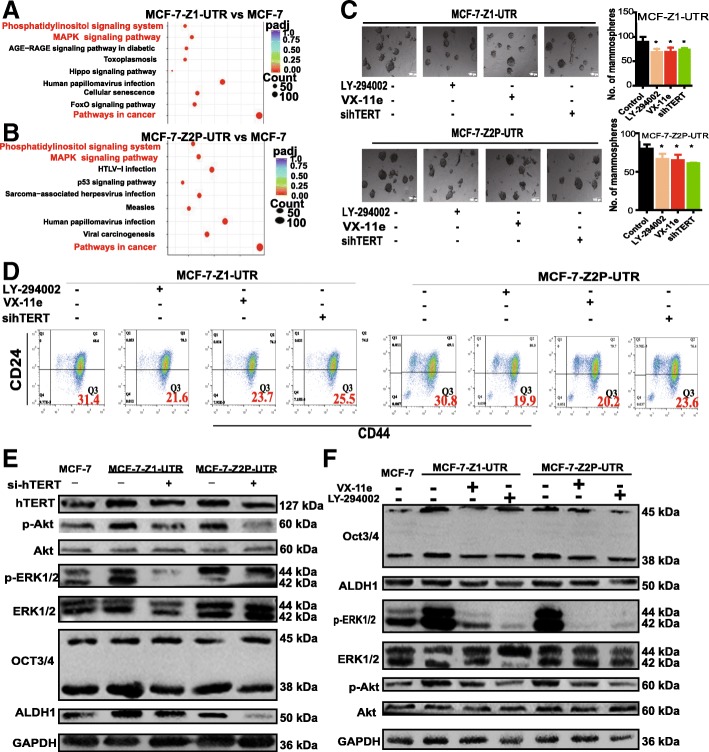
CeRNET_CC promotes the stemness of breast cancer cells partly through the hTERT/PI3K/Akt and ERK1/2 pathways. **a**, **b** Top-ranked functional pathways enriched by genes in MCF-7-Z1-UTR (**a**) and MCF-7-Z2P-UTR (**b**) cells relative to MCF-7 cells. **c** Phase contrast images of mammospheres formed by MCF-7-Z1-UTR or MCF-7-Z2P-UTR cells with LY-294002, VX-11e, or sihTERT treatment (left); spheres were quantified (right). **d** Representative FACS profile of cells described in **c** with CD24^−^ and CD44^+^ markers. **e**, **f** Cells depicted in **c** were subjected to western blot analysis, and the expression of p-Akt/p-ERK1/2 and stemness markers (ALDH1 and OCT3/4) was then detected. The data are presented as the mean ± SD, *n* = 3, *P < 0.05 vs. MCF-7-Z1-UTR or MCF-7-Z2P-UTR

We next sought to explore the mechanisms through which ceRNET_CC promotes the stemness of breast cancer cells. To address this issue, we characterized pathways regulated by CYP4Z1- or CYP4Z2P-3′UTR based on RNA-sequencing data. As expected, we found that the phosphatidylinositol signaling system and MAPK signaling pathways in cancer were some of the most highly upregulated pathways after CYP4Z1- or CYP4Z2P-3′UTR overexpression (Fig. 3a, b). These results were supported by our previous studies showing that ceRNET_CC could act as a subceRNA network for hTERT and promote hTERT expression, thus activating hTERT/PI3K/Akt and ERK1/2 signaling [13, 26]. We then aimed to detect whether ceRNET_CC indeed facilitated the stemness of breast cancer cells through these two pathways. MCF-7-Z1-UTR or MCF-7-Z2P-UTR cells were transfected with siRNA against hTERT (sihTERT) or treated with a PI3K/Akt inhibitor (LY-294002) or ERK1/2 inhibitor (VX-11e), and we then detected the formation of cell spheroids and the CD44^+^/CD24^−^ population. As shown in Fig. 3c, d, the increased cell spheroid formation or CD44^+^/CD24^−^ population induced by CYP4Z1- or CYP4Z2P-3′UTR overexpression was attenuated by sihTERT transfection or by LY-294002 or VX-11e treatment. Additionally, the increased expression of stemness markers induced by CYP4Z1- or CYP4Z2P-3′UTR overexpression was attenuated by sihTERT (Fig. 3e) or by LY-294002 or VX-11e treatment (Fig. 3f) and the knockdown of hTERT or treatment with LY-294002 or VX-11e decreased the p-Akt and p-ERK1/2 levels. Thus, these results demonstrate that ceRNET_CC promotes the stemness of breast cancer cells in a manner dependent on the hTERT/PI3K/Akt and ERK1/2 pathways.

### Transcriptional factor six2 induces the progression of ceRNET_CC by directly regulating the transcription of CYP4Z1 and the pseudogene CYP4Z2P

**Fig. 4:**
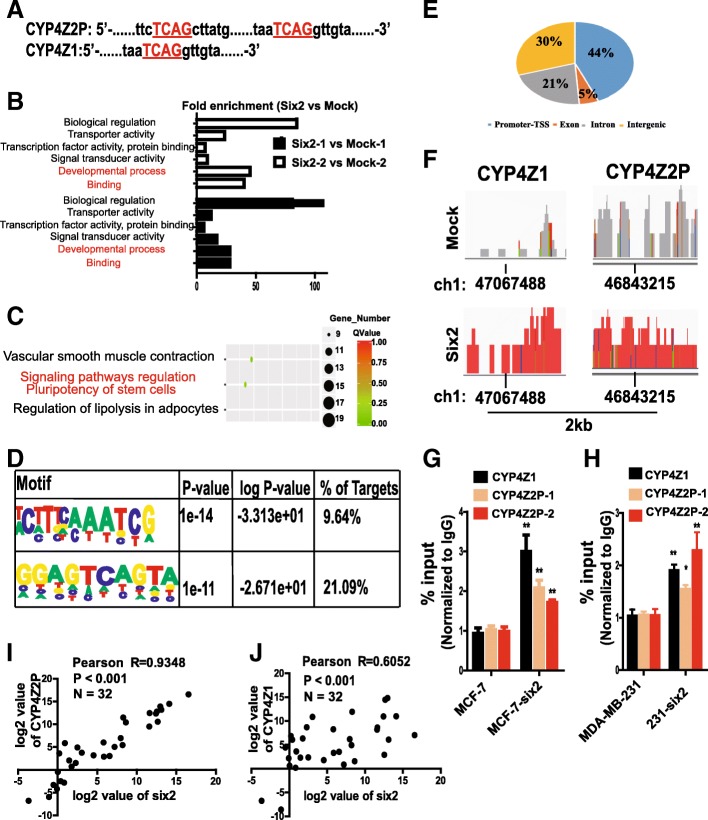
Transcriptional factor six2 induces the progression of ceRNET_CC by directly regulating the transcription of CYP4Z1 and the pseudogene CYP4Z2P. **a** Diagram of the potential binding sites of six2 on the promoters of CYP4Z1 and the pseudogene CYP4Z2P. **b** Functional annotation analysis of genes coordinately activated by six2 overexpression in MCF-7 cells. **c** Enrichment of signaling signatures differentially expressed between MCF-7-six2 and MCF-7 cells based on ChIP-sequencing analysis. **d** The peak motifs of six2-occupied sites. **e** Pie chart showing the percentage of six2-occupied genomic regions that are promoter-TSS, exon, intron, or intergenic. **f** Peak histograms showing six2-occupied CYP4Z1 (left) and pseudogene CYP4Z2P (right) promoters. **g**, **h** ChIP-qRT-PCR analysis of six2 occupancy at selected gene promoters in MCF-7 and MCF-7-six2 cells (**g**) and in MDA-MB-231 and 231-six2 cells (**h**). Error bars represent the SD from three independent experiments (*P < 0.05, **P < 0.01). **i**, **j** Pearson correlation analysis of six2 and the pseudogene CYP4Z2P (**i**) or CYP4Z1 (**j**); log^2^ values of the relative expression levels are presented, *n* = 32, *P* < 0.001

To determine the mechanisms through which ceRNET_CC is regulated, the Genomatix Software Suite v3.10 (https://www.genomatix.de) was used to predict transcriptional factors that could bind to the promoters of CYP4Z1 and CYP4Z2P. We aimed to identify specific transcriptional factors that correlate with tumor progression and tissue development, and six2 attracted our attention because it has been shown to promote breast cancer metastasis [18] and regulate the expansion of the nephron progenitor pool during nephrogenesis, which involves a process similar to CSC formation [35]. We identified one (− 1458 nt) and two putative binding sites (− 1125 nt and − 2716 nt) on the promoters of CYP4Z1 and CYP4Z2P, respectively (Fig. 4a). To confirm the interaction between six2 and CYP4Z1 or CYP4Z2P, MCF-7 cells showing stable overexpression of six2 were constructed by lentivirus infection. We then performed a genome-wide chromatin immunoprecipitation sequencing (ChIP-seq) analysis to identify the six2-bound chromatin regions using an antibody against six2. As shown in Fig. 4b, binding molecular function and developmental process were enriched in six2-overexpressing cells. Importantly, signaling pathways regulating stem cell pluripotency were enriched in six2-overexpressed cells (Fig. 4c). Peak and motif analysis revealed that the 5′-TCAG-3′ motif was highly enriched (*P* = 1e^−11^) (Fig. 4d), and the majority of six2 peaks were located at the promoters/transcription start sites (Fig. 4e). Importantly, six2 occupied the promoters of CYP4Z1 and CYP4Z2P (Fig. 4f). We subsequently performed a ChIP assay for six2 in MCF-7 cells with or without six2 overexpression. Our results indicated that six2 was indeed bound to the promoters of CYP4Z1 and CYP4Z2P and that six2 overexpression increased six2 binding to these promoters (Fig. 4g, h). Additionally, we constructed luciferase reporter vectors containing different regions of the promoters of CYP4Z1 and CYP4Z2P and found that the luciferase activity of the luciferase reporter vectors containing the putative binding sites was enhanced in MCF-7 cells with six2 overexpression (Additional file 9: Figure S3A–C), whereas the activity of luciferase vectors with mutated binding sites or without putative binding sites was unaffected when the binding sites were mutated (Additional file 9: Figure S3B–D). These results indicated that six2 could directly bind to the 5′-TCAG-3′ motif in the promoters of CYP4Z1 and CYP4Z2P. Consistently, the expression levels of CYP4Z2P and CYP4Z1 were significantly increased or decreased by six2 overexpression or knockdown, respectively (Additional file 9: Figure S3E–H). Notably, a qRT-PCR assay of clinical samples showed that both six2 and CYP4Z1 expression and six2 and CYP4Z2P expression were positively correlated in breast cancer tissues (*P* < 0.001, Fig. 4i, j). These results suggest that six2 directly binds to the promoters of CYP4Z1 and CYP4Z2P, thus increasing their expression and activating ceRNET_CC.

### Transcriptional factor six2 promotes the stemness of breast cancer cells

**Fig. 5:**
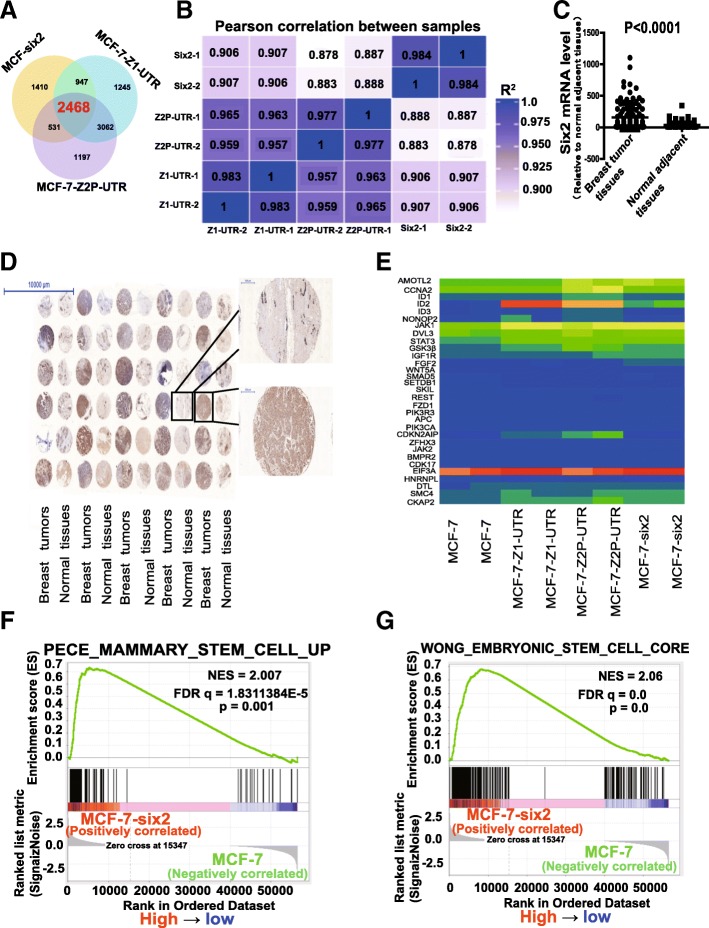
Transcriptional factor six2 promotes the stemness of breast cancer cells in vitro. **a** Venn diagram showing the overlap of genes in MCF-7-Z1-UTR, MCF-7-Z2P-UTR, and MCF-7-six2 cells. **b** Pearson correlation of altered genes in MCF-7-Z1-UTR, MCF-7-Z2P-UTR, and MCF-7-six2 cells. **c** mRNA levels of six2 were examined in breast cancer and normal adjacent tissues via online deposited data (*n* = 1207, the error bars denote the ± SDs, *P* < 0.0001). **d** Six2 expression was evaluated in a tissue microarray including 30 breast cancer tissues and 30 normal adjacent tissues via immunohistochemistry analysis. **e** Heat map showing the mean expression values of genes related to stem cell pluripotency in MCF-7 cells with CYP4Z2P-3′UTR or CYP4Z1-3′UTR or six2 overexpression (fold change > 2 relative to MCF-7 cells, *P* < 0.05). **f**, **g** Enrichment of a stem cell signature according to a GSEA of genes regulated in MCF-7-six2 cells

**Fig. 6:**
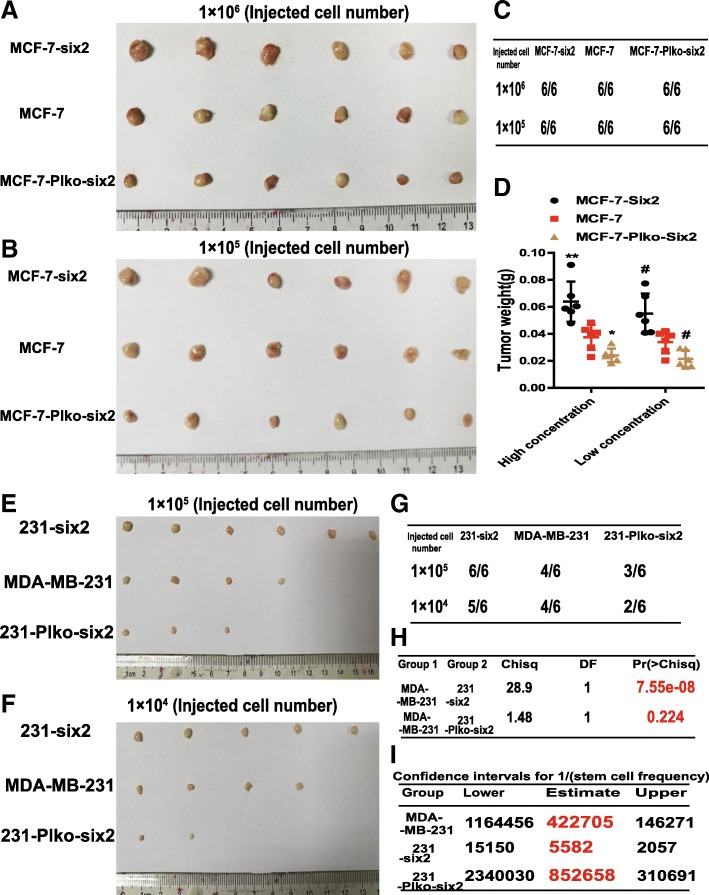
Transcriptional factor six2 promotes the stemness of breast cancer cells in vivo. **a**–**c** Images (**a**, **b**) and number (**c**) of tumors harvested when serially diluted MCF-7, MCF-7-six2, and MCF-7-Plko-six2 were seeded. **d** Weight of tumors harvested when serially diluted cells depicted in **a** were seeded, the data are presented as the means ± SDs, ^*^P < 0.05, ^**^P < 0.01, ^#^*P* < 0.05 vs. MCF-7 group. **e**–**g** Images (**e**, **f**) and number (**g**) of tumors harvested when serially diluted MDA-MB-231, 231-six2, and 231-Plko-six2 were seeded. **h**, **i** Pr values, associated probabilities (**h**), and TIC (tumor-initiating cell) frequencies (**i**) of cells depicted in **e**

Notably, we indeed observed a large overlap and positive correlation between gene expression profiles regulated by CYP4Z1-3′UTR- or CYP4Z2P-3′UTR and six2 in MCF-7 cells (Fig. 5a, b). We next explored whether six2 exerts similar effects on ceRNET_CC. First, six2 expression was examined in breast tumor and normal adjacent tissues via online clinical deposited data (http://www.firebrowse.org/). The results showed that six2 expression was significantly increased in breast tumor tissues (Fig. 5c), and a further tissue microarray analysis exhibited consistent results (Fig. 5d). Additionally, an analysis of different clinical samples (http://hgserver1.amc.nl/cgi-bin/r2/main.cgi) showed that six2 expression was negatively correlated with OS, disease-free survival, and RFS of breast cancer patients (Additional file 10: Figure S4A–F). Notably, we identified a set of gene signatures regulating the pluripotency of stem cells in MCF-7 cells with overexpressing CYP4Z2P-3′UTR, CYP4Z1-3′UTR, and six2, and these genes included AMOTL2, CCNA2, ID1/2, GSK3β, and FGF2 (Fig. 5e). A GSEA revealed that six2 overexpression resulted in the enrichment of gene sets related to embryonic stem cell and mammary stem cell function (Fig. 5f, g), consistent with the CYP4Z1-3′UTR or CYP4Z2P-3′UTR overexpression-mediated changes. As expected, six2 overexpression potentiated sphere formation in MCF-7 cells, whereas the knockdown of six2 exerted the opposite effects (Additional file 11: Figure S5A). Moreover, six2 overexpression increased the CD44^+^/CD24^−^ population, whereas the knockdown of six2 decreased this population (Additional file 11: Figure S5B and S5C). Furthermore, the expression of several pluripotent transcription factors was increased in cells with six2 overexpression and decreased in cells with six2 knockdown (Additional file 11: Figure S5D–G). We further investigated whether six2 could promote the tumor-initiating potential of breast cancer cells in vivo. We compared the capacity of six2-overexpressing or six2-knockdown MCF-7 cells to seed tumors at limiting dilutions. Although all cell lines could form tumors at the densities of 1 × 10^6^ and 1 × 10^5^ cells, the six2-overexpressing cells showed an increase of tumor size and weight, whereas six2-knockdown cells exhibited decreased tumor size and weight (Fig. 6a–d). Furthermore, an in vivo tumorigenic assay was performed with MDA-MB-231 cells after six2 overexpression or knockdown and demonstrated that six2 remarkably attenuated the tumor-initiating potential of MDA-MB-231 cells (Fig. 6e–i). Together, our gain- and loss-of-function assays demonstrate that six2 could promote the stemness of breast cancer cells.

### ceRNET_CC is sufficient and necessary for six2-induced effects

**Fig. 7:**
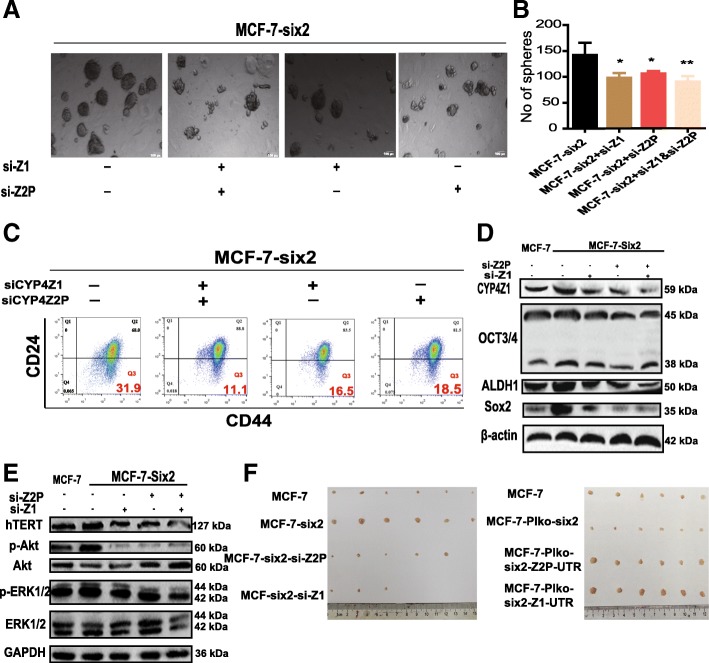
CeRNET_CC is sufficient and necessary for six2-induced effects. **a**, **b** Phase contrast images of mammospheres formed by MCF-7-six2 cells with si-CYP4Z1 or si-CYP4Z2P treatment (**a**) and quantification of spheres (**b**). The data are presented as the means ± SDs, *n* = 3, *P < 0.05, **P < 0.01 vs. MCF-7-six2. **c** Representative FACS profile of cells described in **a** with CD24^−^ and CD44^+^ markers. **d**, **e** Cells depicted in **a** were subjected to western blot analysis and followed by detecting the expression of p-Akt/p-ERK1/2 (**e**) and stemness markers (ALDH1 and OCT3/4) (**d**). **f** Images of tumors harvested when MCF-7, MCF-7-six2, MCF-7-six2-si-Z1, and MCF-7-six2-si-Z2P cells (left) and MCF-7-Plko-six2, MCF-7-Plko-six2-Z1-UTR, and MCF-7-Plko-six2-Z2P-UTR cells (right) were planted

We continued investigating whether the ability of six2 to promote the stemness of breast cancer cells is dependent on ceRNET_CC. MCF-7-six2 cells were transfected with siRNA against CYP4Z1- (si-Z1) or CYP4Z2P-3′UTR (si-Z2P), and the resulting cells were subjected to cell spheroid formation and CD44^+^/CD24^−^ population assays. As shown in Fig. 7a–d, the increased cell spheroid formation, CD44^+^/CD24^−^ population, and stemness marker expression induced by six2 overexpression were nullified by si-Z1 and/or si-Z2P transfection. CYP4Z1- or CYP4Z2P-3′UTR knockdown decreased the hTERT, p-Akt, and p-ERK1/2 levels or even reversed the six2-mediated increase in the hTERT, p-Akt, and p-ERK1/2 levels (Fig. 7e). Notably, co-transfection with si-Z1 and si-Z2P exerted additive effects. Importantly, we constructed MCF-7-six2 cells with CYP4Z1 or CYP4Z2P knockdown, designated as MCF-7-six2-si-Z1 and MCF-7-six2-si-Z2P cells, respectively, and MCF-7-Plko-six2 cells with CYP4Z1- or CYP4Z2P-3′UTR overexpression, denoted as MCF-7-Plko-six2-Z1-UTR and MCF-7-Plko-six2-Z2P-UTR cells, respectively. An in vivo tumorigenic assay showed that six2 overexpression increased the tumor-initiation capacity of MCF-7 cells, an effect that was fully inhibited by CYP4Z1 or CYP4Z2P knockdown, and the decreased tumor-initiation capacity of MCF-7 cells with six2 knockdown was rescued by CYP4Z1- or CYP4Z2P-3′UTR overexpression (Fig. 7f).

Additionally, we detected whether the six2-mediated promotion of breast cancer cell stemness occurs through the downstream effectors of the ceRNA network between CYP4Z1 and the pseudogene CYP4Z2P and the hTERT/PI3K/Akt and ERK1/2 pathways. To address this issue, we characterized pathways regulated by six2 based on RNA-sequencing data. As expected, we found that PI3K-Akt signaling pathway, phosphatidylinositol signaling system, and signaling pathways regulating pluripotency of cells and breast cancer were among the pathways most highly upregulated by six2 overexpression (Additional file 12: Figure S6A). As expected, the increased cell spheroid formation or CD44^+^/CD24^−^ population induced by six2 overexpression was attenuated by sihTERT transfection or by LY-294002 or VX-11e treatment (Additional file 12: Figure S6B–D). Moreover, the increased expression of stemness markers induced by six2 overexpression was attenuated by sihTERT (Additional file 12: Figure S6E), or by LY-294002 or VX-11e treatment (Additional file 12: Figure S6F). Furthermore, the overexpression or knockdown of six2 increased or decreased the expression of hTERT, p-ERK1/2, and p-AKT in MDA-MB-231 cells, respectively (Additional file 12: Figure S6G). Consistent with these results, the knockdown of CYP4Z1, CYP4Z2P, or hTERT or the treatment with LY-294002 or VX-11e reduced the ability of six2 overexpression to promote hTERT expression, PI3K/Akt signaling, ERK1/2 signaling, or the expression of cell stemness markers in MDA-MB-231 cells (Additional file 12: Figure S6H). Notably, we found that the overexpression of CYP4Z1- or CYP4Z2P-3′UTR rescued the six2 knockdown-mediated inhibition of breast cancer cell stemness, characterized by increasing cell spheroid formation (Additional file 13: Figure S7A and S7B), CD44^+^/CD24^−^ population (Additional file 13: Figure S7C), and expression of stemness markers and reactivation of the PI3K/Akt and ERK1/2 signaling (Additional file 13: Figure S7D–G). Thus, these results demonstrate that six2 promotes breast cancer cell stemness dependent on ceRNET_CC.

### Six2-mediated regulation of ceRNET_CC renders breast cancer cells resistant to adriamycin treatment by promoting cell stemness

**Fig. 8:**
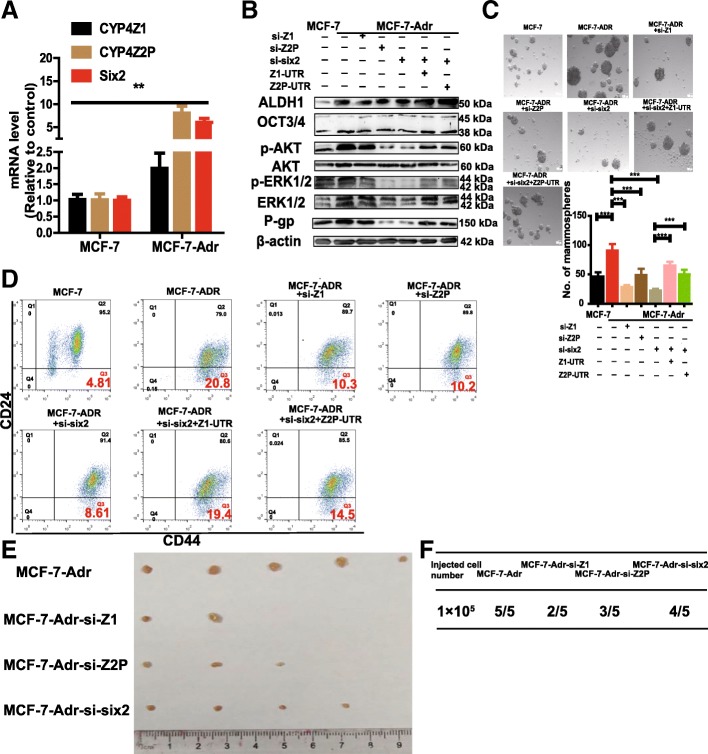
Six2-mediated regulation of ceRNET_CC renders breast cancer cell resistance to adriamycin treatment by promoting cell stemness. **a** CYP4Z2P, CYP4Z1, and six2 expression were detected in MCF-7 and MCF-7-Adr cells by qRT-PCR. **b** MCF-7-Adr cells were transfected with si-CYP4Z1, si-CYP4Z2P, or si-six2 plus Z1-UTR, or Z2P-UTR and followed by detecting the expression of p-Akt/p-ERK1/2, P-gp, and stemness markers (ALDH1 and OCT3/4). **c** Phase contrast images of mammospheres formed by cells depicted in **b**, and spheres were quantified. The data are presented as the means ± SDs, *n* = 3, **P < 0.001 vs. MCF-7. **d** Representative FACS profile of cells described in **b** with CD24^−^ and CD44^+^ markers. **e**, **f** Images (**e**) and number (**f**) of tumors harvested when cells described in **b** were seeded

We have established the ability of the six2/ceRNET_CC axis to promote CSC traits in breast cancer cells. As conferring CSC traits have been confirmed to endow chemoresistance to tumor cells [36], we speculated whether this regulatory axis could decrease adriamycin sensitivity in breast cancer cells by promoting cell stemness. First, the expression of six2, CYP4Z1, and the pseudogene CYP4Z2P was examined in MCF-7 and adriamycin-resistant MCF-7 (MCF-7-Adr) cells via qRT-PCR assay, and the expression was shown to be significantly increased in MCF-7-Adr cells (Fig. 8a). Furthermore, IC_50_ values were determined in MCF-7 and MDA-MB-231 cells with CYP4Z1- or CYP4Z2P-3′UTR overexpression or knockdown, or with six2 overexpression or knockdown, and we found that overexpression of CYP4Z1- or CYP4Z2P-3′UTR or six2 increased the IC_50_ values of adriamycin, while knockdown decreased the IC_50_ values in both MCF-7 and MDA-MB-231 cells (Table 1). MCF-7-Adr cells displayed higher expression of P-gp (a multidrug resistance protein) and pluripotent transcription factors and hyperactivation of PI3K/Akt and ERK1/2 signaling, both of which were attenuated by the knockdown of CYP4Z1- or CYP4Z2P-3′UTR or six2, and the overexpression of CYP4Z1- or CYP4Z2P-3′UTR rescued the attenuation mediated by six2 knockdown (Fig. 8b). Additionally, the knockdown of CYP4Z1- or CYP4Z2P-3′UTR or six2 decreased the spheroid formation ability (Fig. 8c) and the CD44^+^/CD24^−^ population in MCF-7-Adr cells (Fig. 8d), and the decrease induced by six2 knockdown was rescued by the overexpression of CYP4Z1- or CYP4Z2P-3′UTR. Notably, the knockdown of CYP4Z1- or CYP4Z2P-3′UTR or six2 enhanced adriamycin sensitivity, evidenced by the decrease of IC_50_ values of adriamycin (Table 1). Consistently, the knockdown of CYP4Z1- or CYP4Z2P-3′UTR or six2 impaired the tumor-initiating potential of MCF-7-Adr cells (Fig. 8e, f). Thus, our results indicate that the six2/ceRNET_CC regulatory axis attenuates adriamycin sensitivity by promoting the stemness of breast cancer cells (Additional file 14: Figure S8).

**Table 1:** The IC_50_ value of adriamycin in different cells

Cell name	IC_50_ value ((μM)	*P* value
MCF-7	3.637 ± 0.028	
MCF-7-Z1-UTR	6.496 ± 0.017	**
MCF-7-Z2P-UTR	6.49 ± 0.013	**
MCF-7-six2	9.31 ± 0.009	**
MCF-7-Plko-Z1	0.4349 ± 0.024	**
MCF-7-Plko-Z2P	0.546 ± 0.029	**
MCF-7-Plko-six2	0.4871 ± 0.017	**
MDA-MB-231	2.853 ± 0.034	
231-Z1-UTR	4.208 ± 0.015	**
231-Z2P-UTR	4.081 ± 0.011	**
231-Plko-Z1	0.6296 ± 0.033	**
231-Plko-Z2P	0.7747 ± 0.036	**
231-Plko-six2	0.3066 ± 0.030	**
MCF-7-Adr	41.12 ± 5.93	
MCF-7-Adr-Plko-Z1	19.87 ± 3.33	**
MCF-7-Adr-Plko-Z2P	11.33 ± 1.68	**
MCF-7-Adr-Plko-six2	6.971 ± 1.10	**
MCF-7-Adr-Plko-six2-Z1-UTR	10.8 ± 1.31	##
MCF-7-Adr-Plko-six2-Z2P-UTR	9.235 ± 1.12	##

**p < 0.01 vs MCF-7 or MDA-MB-231 or MCF-7-Adr cells, ^##^*p* < 0.01 vs MCF-7-Plko-six2 cells

## Discussion

The pseudogene CYP4Z2P was first identified in 2004 by Rieger et al., who showed that the expression of both the pseudogene CYP4Z2P and its functional gene CYP4Z1 were specifically increased in breast cancer [37]. Further study indicated that the expression of CYP4Z1 and the pseudogene CYP4Z2P was associated with PIK3CA mutations in ERalpha-positive breast cancer [38]. These effects suggest that CYP4Z1 and the pseudogene CYP4Z2P might be involved in breast cancer progression. Our previous studies established that ceRNET_CC promotes angiogenesis [26], confers tamoxifen resistance [39], and serves as an anti-apoptotic factor in breast cancer cells [13]. In the present study, we further determined its role and underlying molecular mechanism in regulating the stemness of breast cancer, and we obtained the following findings: (1) the expression levels of CYP4Z1 and the pseudogene CYP4Z2P were higher in breast cancer sphere cells and adriamycin-resistant MCF-7-Adr cells; (2) RNA-sequencing and pathway analysis combined with in vitro and in vivo experiments indicated that ceRNET_CC promoted the stemness of breast cancer cells by activating the PI3K/Akt and ERK1/2 signaling pathways, both of which are critically involved in pro-survival and pro-stem-cell maintenance [40–42]; (3) ChIP-sequencing and a ChIP assay confirmed that transcriptional factor six2 directly bound to the 5′-TCAG-3′ motif in the promoters of CYP4Z1 and CYP4Z2P; (4) six2 expression was increased in breast cancer tissues and facilitated the progression of ceRNET_CC, thereby enhancing the stemness of breast cancer cells; (5) the six2-mediated regulation of ceRNET_CC contributed to adriamycin resistance by regulating the stemness of breast cancer cells, and this effect was due at least in part to the activation of PI3K/Akt and ERK1/2 signaling pathways; and (6) the expression of both six2 and CYP4Z1 was negatively correlated with the OS of breast cancer patients and positively correlated with the expression of the stemness marker Nanog, and furthermore, six2 and CYP4Z1 expression was positively correlated in breast cancer tissues. These findings provide the first insights into the roles and molecular mechanism of the six2-mediated regulation of ceRNET_CC, which promotes stemness and chemoresistance in breast cancer.

A previous study indicated that tumor cells with higher expression of hTERT displayed stronger stemness characteristics [43]. This work and our previous work have suggested that ceRNET_CC acts as a subceRNA network for hTERT [13], which fully supports the role of ceRNET_CC in promoting the stemness of breast cancer cells. Additionally, combined with the current work showing that ceRNET_CC attenuates adriamycin sensitivity in estrogen receptor (ER)-positive and ER-negative breast cancer, our previous study showed that ceRNET_CC confers tamoxifen resistance in ER-positive breast cancer [39]. Additionally, KEGG pathway enrichment showed that endocrine resistance and EGFR tyrosine kinase inhibitor resistance were enriched in MCF-7 cells with CYP4Z1- or CYP4Z2P-3′UTR or six2 overexpression (data not shown). These results strongly support ceRNET_CC as a CSC-related marker because CSCs contribute to drug resistance. Nevertheless, we investigated adriamycin sensitivity in this work because it is one of the first-line drugs used for chemotherapy in breast cancer patients, and it remains unclear whether six2/ceRNET_CC contributes to resistance to other drugs. Notably, due to the relatively lower stemness of MCF-7 cells and higher stemness of MDA-MB-231 and MCF-7-Adr cells [7, 44], we mostly chose to overexpress CYP4Z1-3′UTR or CYP4Z2P-3′UTR or six2 in MCF-7 cells and to knock these factors down in MDA-MB-231 and MCF-7-Adr cells.

Importantly, current treatments for triple-negative breast cancer (TNBC), which is the deadliest form of breast cancer, rely mainly on chemotherapy because no targeted therapies are currently approved for TNBC [45], raising the potential for ceRNET_CC-targeted therapies in TNBC. Furthermore, a previous study demonstrated that six2 could bind to the E-cadherin promoter and enhance its methylation levels in breast cancer cells [18]. However, the exact six2 binding sites remained unclear. Here, a ChIP-sequencing assay indicated that six2 specifically bound to the 5′-TCAG-3′ motif. This is the first study elucidating the binding sites of six2 in breast cancer. However, it remains unclear whether this is a common phenomenon. GSEA analysis indicated that *ACCAAAG_MIR9*, *CATGTAA_MIR496*, *GTTATAT_MIR410*, *ACATATC_MIR190*, *IKEDA_MIR30_TARGETS_UP*, and *3′UTR-mediated translational regulation* were enriched in MCF-7-six2 cells (data not shown). These five miRNAs were predicted to bind to the 3′UTR of both CYP4Z1 and the pseudogene CYP4Z2P, and KEGG enrichment showed that miRNAs in cancers were also enriched in MCF-7-six2 cells (data not shown), which suggested that six2 might regulate ceRNET_CC through the 3'UTR and shared miRNAs of CYP4Z1 and the pseudogene CYP4Z2P. These findings should be explored in future work. A previous study indicated that six2 defines and regulates a multipotent self-renewing nephron progenitor population throughout mammalian kidney development [17], and recent work has shown that normal cells and CSCs might share regulatory mechanisms for maintaining self-renewing capacity and resisting differentiation [46]. Moreover, the YAP1/six2 axis is required for DDX3-mediated tumor aggressiveness and cetuximab resistance in KRAS wild-type colorectal cancer [47]. Notably, the WNT signaling pathway, Jak-STAT signaling pathway, and FoxO signaling pathway, which are involved in regulating cell stemness [48–50], were enriched in MCF-7 cells with CYP4Z1- or CYP4Z2P-3′UTR or six2 overexpression (data not shown). Although we cannot exclude the possibility that six2 may still function through additional signaling pathways to regulate the stemness of breast cancer cells, our study firmly establishes the critical roles of six2-mediated regulation of ceRNET_CC in these processes.

Notably, six2 protein levels were gradually increased by adriamycin treatment in a concentration-dependent manner (data not shown), suggesting that six2 overexpression and adriamycin resistance might form a positive feedback loop in breast cancer cells. Despite efforts to develop chemotherapies for killing CSCs over the past decades and evidence of early success [43, 51, 52], there have been significant setbacks, presumably due to limited effectiveness in late-stage clinical trials. In addition to toxicity and side effects, the reasons for the setbacks could also be the lack of predictive biomarkers for patients. Our finding that the six2/ceRNET_CC regulatory axis promotes the stemness of breast cancer provides putative targets for the development of new strategies for targeting and compromising the maintenance of breast cancer stemness, and we hypothesize that a gene expression signature comprising all three of these genes will predict chemotherapeutic sensitivity in breast cancer patients.

## Additional files

Additional file 1: **Table S1**. Sequences of primers used for qRT-PCR in this study. (DOCX 17 kb)

Additional file 2: **Table S2.** Primary antibodies used in this study. (DOC 35 kb)

Additional file 3: **Table S3.** Sequences of primers used for ChIP qRT-PCR in this study. (DOCX 16 kb)

Additional file 4: **Table S4.** Sequences of siRNA against specific target in this study (DOC 32 kb)

Additional file 5: **Table S5.** Sequences of primers used for plasmid constructions. (DOC 40 kb)

Additional file 6: **Table S6.** Sequences of primers used for Luciferase reporter assay. (DOC 46 kb)

Additional file 7: **Figure S1.** CeRNET_CC promotes the stemness of MCF-7 cells in vitro*.* (A) The expression of CYP4Z2P and CYP4Z1 in MCF-7 and MCF-7-tumorsphere cells was detected by qRT-PCR. (B and C) The infection efficiency of MCF-7 cells with CYP4Z1- or CYP4Z2P-3′UTR stable overexpression (B) or knockdown (C) was detected by qRT-PCR. (D) Phase contrast images of mammospheres formed by stable expression cells depicted in B and C and quantification of spheres. (E) Representative FACS profile of cells described in B with CD24^−^ and CD44^+^ markers. (F and G) The mRNA and protein expression of stemness markers (ALDH1, SOX2, OCT4 and Nanog) in cells described in B and C were examined by qRT-PCR and western blot analysis, respectively. The data are presented as the means ± SDs, *n* = 3, *P < 0.05, **P < 0.01, ***P < 0.001 vs. MCF-7. (PDF 5600 kb)

Additional file 8: **Figure S2.** CeRNET_CC promotes the stemness of MDA-MB-231 cells in vitro*.* (A and B) The infection efficiency of MDA-MB-231 cells with CYP4Z1- (A) or CYP4Z2P-3′UTR (B) stable knockdown was detected by qRT-PCR. (C) Phase contrast images of mammospheres formed by stable expression cells depicted in A and B and quantification of spheres. (D) Representative FACS profile of cells described in A and B with CD24^−^ and CD44^+^ markers. (E and F) The mRNA and protein expression of stemness markers (ALDH1, SOX2, OCT4, and Nanog) in cells described in A and B. (G) Pearson correlation analysis of the expression of CYP4Z1 and Nanog in basal-like breast cancer (*n* = 252, *P* < 0.01). (H) Pearson correlation analysis of the expression of the pseudogene CYP4Z2P and Nanog in basal-like breast cancer (*n* = 144, *P* < 0.01). (PDF 3450 kb)

Additional file 9: **Figure S3.** Transcriptional factor six2 promotes the expression of CYP4Z1 and the pseudogene CYP4Z2P. (A) Computational analysis of the CYP4Z2P and CYP4Z1 promoters showed potential binding sites for six2. (B and C) Fragments of the CYP4Z2P and CYP4Z1 promoters were cloned into the luciferase reporter vector pGL3. MCF-7 cells were co-transfected with six2 and the luciferase constructs or the control construct. 72 h later, luciferase activity was measured. (D) Relative luciferase activity was detected in MCF-7 cells co-transfected with the six2 overexpression vector and CYP4Z1 and CYP4Z2P promoter vectors with six2 binding sites or mutated six2 binding sites. (E and F) The expression of CYP4Z2P, CYP4Z1, and six2 in MCF-7-six2 (E) and 231-six2 (F) cells was examined by qRT-PCR. (G and H) CYP4Z1 protein expression in MCF-7-six2 (G) and MCF7-Plko-six2 (H) cells was detected by western blot. The data were presented as the means ± SDs, *n* = 3, **P < 0.01 vs. control or MCF-7. (PDF 1270 kb)

Additional file 10: **Figure S4.** The correlation between six2 expression and the survival of breast cancer patients. (A) KM-plotter survival curves showed the disease-free survival probability of patients separated into low and high six2 levels. (B) KM-plotter survival curves showed the OS survival probability of patients separated into low and high six2 level. (C and F) KM-plotter survival curves showed the RFS probability of patients separated into low and high six2 levels (http://hgserver1.amc.nl/cgi-bin/r2/main.cgi). (PDF 576 kb)

Additional file 11: **Figure S5.** Transcriptional factor six2 promotes the stemness of breast cancer cells in vitro. (A) Phase contrast images of mammospheres formed by MCF-7 cells with or without six2 overexpression or knockdown and MDA-MB-231 cells with or without six2 knockdown; spheres were quantified. (B and C) Representative FACS profile of MCF-7 cells with or without six2 overexpression (B) and MDA-MB-231 cells (C) with or without six2 knockdown, with the CD24^−^ and CD44^+^ markers. (D and E) The mRNA expression of stemness markers (ALDH1, SOX2, OCT4, and Nanog) in MCF-7 (D) or MDA-MB-231(E) cells with six2 stable overexpression or knockdown was detected by qRT-PCR. (F and G) Cells depicted in D and E were subjected to western blot analysis and followed by detecting the expression of six2 and stemness markers (ALDH1, SOX2, and OCT3/4). The data are presented as the mean ± SD, n = 3, *P < 0.05, **P < 0.01, ***P < 0.001 vs. MCF-7 or MDA-MB-231. (PDF 4250 kb)

Additional file 12: **Figure S6.** Transcriptional factor six2 promotes the stemness of breast cancer cells partly through the hTERT/PI3K/Akt and ERK1/2 pathways. (A) Functional annotation analysis of genes coordinately activated by six2 overexpression. (B and C) Phase contrast images of mammospheres formed by MCF-7-six2 cells with LY-294002, VX-11e, or sihTERT treatment (B), and spheres were quantified (C). The data are presented as the means ± SDs, n = 3, *P < 0.05 vs. MCF-7-six2. (D) Representative FACS profile of cells described in B with CD24^−^ and CD44^+^ markers. (E and F) Cells depicted in B were subjected to western blot analysis and followed by detecting the expression of p-Akt/p-ERK1/2 and stemness markers (ALDH1 and OCT3/4). (G) MDA-MB-231 cells with six2 stable overexpression or knockdown were subjected to western blot analysis and followed by detecting the expression of p-Akt/p-ERK1/2 and hTERT. (H) 231-six2 cells with LY-294002, VX-11e, or sihTERT, or si-Z1, or si-Z2P treatment were subjected to western blot analysis and followed by detecting the expression of p-Akt/p-ERK1/2 and stemness markers (ALDH1 and OCT3/4). (PDF 5040 kb)

Additional file 13: **Figure S7.** CeRNET_CC is sufficient and necessary for six2-induced effects. (A and B) Phase contrast images of mammospheres formed by MCF-7-Plko-six2 cells with Z1-UTR or Z2P-UTR overexpression (A); spheres were quantified (B). The data are presented as the means ± SDs, *n* = 3, *P < 0.05, **P < 0.01 vs. MCF-7 or MCF-7-six2. (C) Representative FACS profile of cells described in A with CD24^−^ and CD44^+^ markers. (D–G) MCF-7-Plko-six2 (D and F) and 231-Plko-six2 (E and G) cells with Z1-UTR, Z2P-UTR (D and E), or hTERT (F and G) overexpression were subjected to western blot analysis and followed by detecting the expression of p-Akt/p-ERK1/2 and stemness markers (ALDH1 and OCT3/4). The data were presented as the mean ± SD, *n* = 3, *P < 0.05, **P < 0.01 vs. MCF-7 or MCF-7-six2. (PDF 3760 kb)

Additional file 14: **Figure S8.** Proposed model in which transcriptional factor six2-mediated regulation of ceRNET_CC is responsible for breast CSC formation and thus drug resistance. Transcriptional factor six2 induces the progression of ceRNET_CC by directly binding to the promoters of CYP4Z1 and the pseudogene CYP4Z2P. This six2/ceRNET_CC regulatory axis results in breast CSC progression and thus enhances drug sensitivity. (TIF 242 kb)

## Abbreviations

cDNA: Complementary DNA
ceRNA: Competitive endogenous RNA
ceRNET_CC: ceRNA network between CYP4Z1 and pseudogene CYP4Z2P
ChIP: Chromatin immunoprecipitation
CSCs: Cancer stem cells
FBS: Fetal bovine serum
GEO: Gene Expression Omnibus
GSEA: Gene set enrichment analysis
NC: Negative control
NSCLC: Non-small-cell lung cancer
OS: Overall survival
qRT-PCR: Quantitative real-time PCR
RFS: Relapse-free survival
siRNA: Small interfering RNA
STR: Short tandem repeat
