# Correction to: Transcriptional factor six2 promotes the competitive endogenous RNA network between CYP4Z1 and pseudogene CYP4Z2P responsible for maintaining the stemness of breast cancer cells

**DOI:** 10.1186/s13045-019-0809-3

**Published:** 2019-10-24

**Authors:** Lufeng Zheng, Qianqian Guo, Chenxi Xiang, Shijia Liu, Yuzhang Jiang, Lanlan Gao, Haiwei Ni, Ting Wang, Qiong Zhao, Hai Liu, Yingying Xing, Yaohui Wang, Xiaoman Li, Tao Xi

**Affiliations:** 1 Jiangsu Key Laboratory of Carcinogenesis and Intervention, School of Life Science and Technology, China Pharmaceutical University, 24 Tong Jia Xiang, Nanjing, 210009 China; 2 Jiangsu Key Laboratory for Pharmacology and Safety Evaluation of Chinese Materia Medica, School of Pharmacy, Nanjing University of Chinese Medicine, Nanjing, 210023 China; 3 Department of Pathology, The Affiliated Hospital of Xuzhou Medical University, Xuzhou, 221002 Jiangsu China; 4 Department of Pharmacy, Jiangsu Province Hospital of TCM, Nanjing, 210023 China; 5 Department of Clinical Laboratory, Huai An First People’s Hospital, Huai An, 223300 China; 6 Department of Pathology, Jiangsu Province Hospital of TCM, Nanjing, 210023 China

**Correction to: J Hematol Oncol (2019) 12:23**

**https://doi.org/10.1186/s13045-019-0697-6**

The original article [1] contained an error in Fig. 7c whereby the same flow image was accidentally misused for the second and fourth group. The correct version of Fig. 7c can be viewed below together with the rest of Fig. 7.

**Fig. 7:**
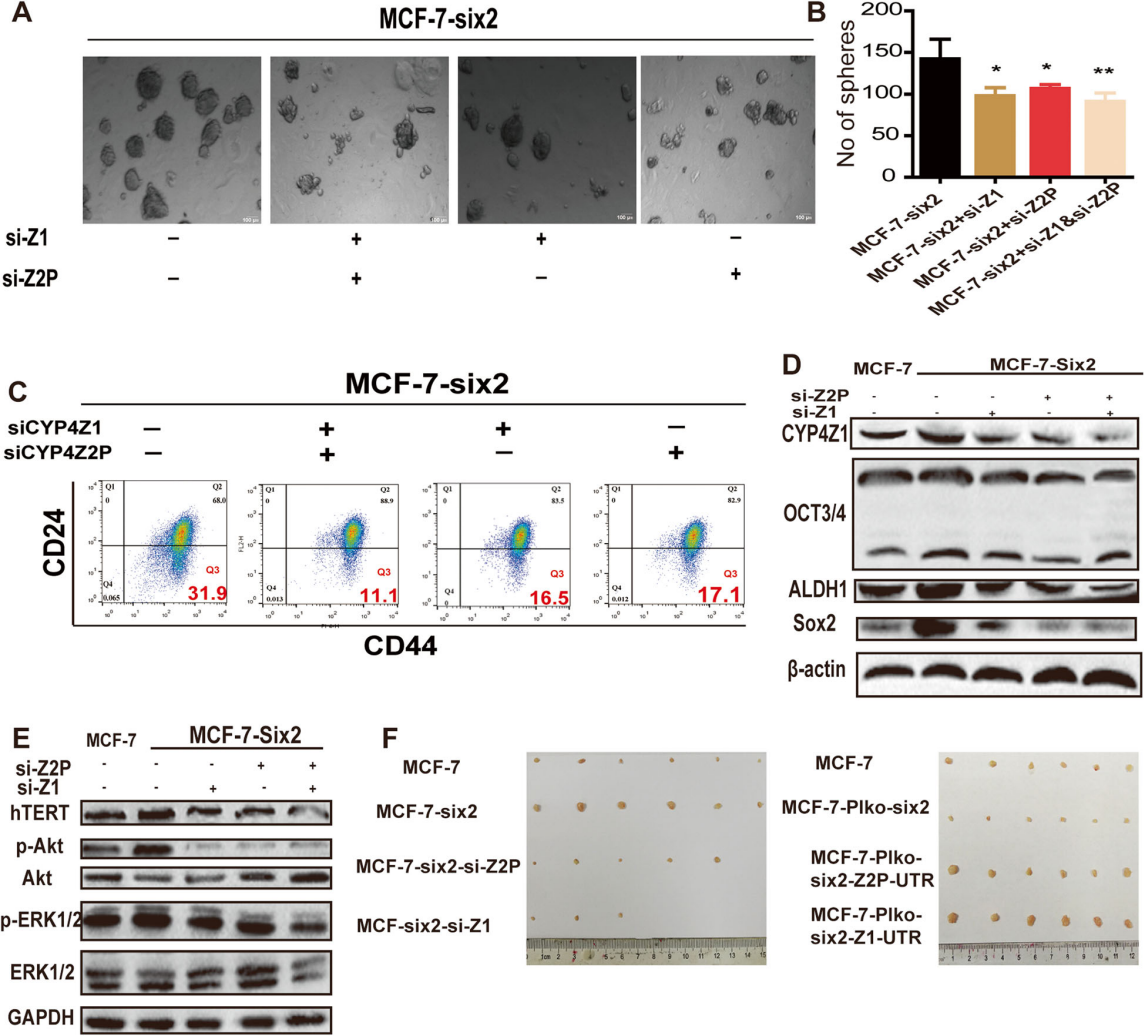
CeRNET_CC is sufficient and necessary for six2-induced effects. **a**, **b** Phase contrast images of mammospheres formed by MCF-7-six2 cells with si-CYP4Z1 or si-CYP4Z2P treatment (**a**) and quantification of spheres (**b**). The data are presented as the means ± SDs, *n* = 3, *P < 0.05, **P < 0.01 vs. MCF-7-six2. **c** Representative FACS profile of cells described in **a** with CD24− and CD44+ markers. **d**, **e** Cells depicted in **a** were subjected to western blot analysis and followed by detecting the expression of p-Akt/p-ERK1/2 (**e**) and stemness markers (ALDH1 and OCT3/4) (**d**). **f** Images of tumors harvested when MCF-7, MCF-7-six2, MCF-7-six2-si-Z1, and MCF-7-six2-si-Z2P cells (left) and MCF-7-Plko-six2, MCF-7-Plko-six2-Z1-UTR, and MCF-7-Plko-six2-Z2P-UTR cells (right) were planted
