# Supplementary material for: Transcriptional factor six2 promotes the competitive endogenous RNA network between CYP4Z1 and pseudogene CYP4Z2P responsible for maintaining the stemness of breast cancer cells
Source: J Hematol Oncol. 2019 Mar 4;12:23. doi: 10.1186/s13045-019-0697-6 (PMC6399913; doi:10.1186/s13045-019-0697-6)
Supplement: Supplementary file 1 — Table S1. Sequences of primers used for qRT-PCR in this study. (DOCX 17 kb) [file 13045_2019_697_MOESM1_ESM.docx]

**Additional file 1: Table S1. Sequences of primers used for qRT-PCR in this study**

| Name |  | Sequences |
| --- | --- | --- |
| CYP4Z1-3'UTR-F | Forward (5’-3’) | CTACCAAAGGAAGAACAAAAGGATA |
|  | Reverse (5’-3’) | GGGGAAAGATAACTGAGAATAAAGC |
| CYP4Z2P-3'UTR | Forward (5’-3’) | CTTTCCAGATGGACGCTCCTTACCT |
|  | Reverse (5’-3’) | CCAGCAAGGAAATTAGAATTACTTAATCC |
| CYP4Z1 | Forward (5’-3’) | CTTTCCAGATGGACGCTCCTTACCT |
|  | Reverse (5’-3’) | GGCAAAATGCTGCCCAATGCAGTTC |
| CYP4Z2P | Forward (5’-3’) | CTTTCCAGATGGACGCTCCTTACCT |
|  | Reverse (5’-3’) | CCAGCAAGGAAATTAGAATTACTTAATCC |
| Six2 | Forward (5’-3’) | AAGGCACACTACATCGAGGC |
|  | Reverse (5’-3’) | CACGCTGCGACTCTTTTCC |
| ALDH1 | Forward (5’-3’) | AGCCTTCACAGGATCAACAGA |
|  | Reverse (5’-3’) | GTCGGCATCAGCTAACACAA |
| Nanog | Forward (5’-3’) | GCAGGCAACTCACTTTATCC |
|  | Reverse (5’-3’) | CCCACAAATCACAGGCATAG |
| Oct4 | Forward (5’-3’) | AGCGATCAAGCAGCGACTA |
|  | Reverse (5’-3’) | GGAAAGGGACCGAGGAGTA |
| Sox2 | Forward (5’-3’) | CATCACCCACAGCAAATGAC |
|  | Reverse (5’-3’) | CAAAGCTCCTACCGTACCACT |
| GAPDH | Forward (5’-3’) | CTTAGTTGCGTTACACCCTTTCTTG |
|  | Reverse (5’-3’) | CTGTCACCTTCACCGTTCCAGTTT |
