# Supplementary material for: Transcriptional factor six2 promotes the competitive endogenous RNA network between CYP4Z1 and pseudogene CYP4Z2P responsible for maintaining the stemness of breast cancer cells
Source: J Hematol Oncol. 2019 Mar 4;12:23. doi: 10.1186/s13045-019-0697-6 (PMC6399913; doi:10.1186/s13045-019-0697-6)
Supplement: Supplementary file 2 — Table S2. Primary antibodies used in this study. (DOC 35 kb) [file 13045_2019_697_MOESM2_ESM.doc]

**Additional file 2: Table S2. Primary antibodies used in this study.**

| Antigens | Manufacturer | Application |
| --- | --- | --- |
| Six2 | proteintech | 1:1000 for WB |
| CYP4Z1 | proteintech | 1:1000 for WB |
| ALDH1A1 | proteintech | 1:1000 for WB |
| SOX2 | Santa Cruz | 1:1000 for WB |
| Nanog | Santa Cruz | 1:1000 for WB |
| OCT3/4 | proteintech | 1:1000 for WB |
| P-gp | proteintech | 1:1000 for WB |
| p-ERK1/2(Thr202/Tyr204) | Wanleibio (Shenyang, China) | 1:1000 for WB |
| ERK1/2 | Wanleibio | 1:1000 for WB |
| p-AKT-s473 | proteintech | 1:1000 for WB |
| AKT | Wanleibio | 1:1000 for WB |
| β-actin | yifeixue | 1:5000 for WB |
| GAPDH | proteintech | 1:2000 for WB |
