# Supplementary material for: Transcriptional factor six2 promotes the competitive endogenous RNA network between CYP4Z1 and pseudogene CYP4Z2P responsible for maintaining the stemness of breast cancer cells
Source: J Hematol Oncol. 2019 Mar 4;12:23. doi: 10.1186/s13045-019-0697-6 (PMC6399913; doi:10.1186/s13045-019-0697-6)
Supplement: Supplementary file 3 — Table S3. Sequences of primers used for ChIP qRT-PCR in this study. (DOCX 16 kb) [file 13045_2019_697_MOESM3_ESM.docx]

**Additional file 3: Table S3. Sequences of primers used for ChIP qRT-PCR in this study**

| Name |  | Sequences |
| --- | --- | --- |
| CYP4Z1 | Forward (5’-3’) | CTCCTTTGTGTTTATGAGAGACCTG |
|  | Reverse (5’-3’) | ATGTTTTCCCATTTGTTTGTGTTAT |
| CYP4Z2P(1125) | Forward (5’-3’) | ATTTTCTCCTCTTACCTCCTTGTGG |
|  | Reverse (5’-3’) | ACCTAAAAAACCCTAAAGTCTCTGC |
| CYP4Z2P(2716) | Forward (5’-3’) | TTGTGGGAAAGATAACTGAGAATAA |
|  | Reverse (5’-3’) | ATAACTTTGGATACTTCTGACTGGT |
