# Supplementary material for: Transcriptional factor six2 promotes the competitive endogenous RNA network between CYP4Z1 and pseudogene CYP4Z2P responsible for maintaining the stemness of breast cancer cells
Source: J Hematol Oncol. 2019 Mar 4;12:23. doi: 10.1186/s13045-019-0697-6 (PMC6399913; doi:10.1186/s13045-019-0697-6)
Supplement: Supplementary file 4 — Table S4. Sequences of siRNA against specific target in this study (DOC 32 kb) [file 13045_2019_697_MOESM4_ESM.doc]

**Additional file 4: Table S4. Sequences of siRNA against specific target in this study.**

| Name |  | Sequences |
| --- | --- | --- |
| six2 siRNA | Sense (5’-3’) | GCGAGCUCUACAAGAUCCUTT |
|  | Anti-Sense (5’-3’) | AGGAUCUUGUAGCUCGCTT |
| CYP4Z1 siRNA | Sense (5’-3’) | CAUUACCUUUCCAGAUGGATT |
|  | Anti-Sense (5’-3’) | UCCAUCUGGAAAGGUAAUGTT |
| CYP4Z2P siRNA | Sense (5’-3’) | CCACUCAGUAUCUGCAUUATT |
|  | Anti-Sense (5’-3’) | UAAUGCAGAUACUGAGUGGTT |
| hTERT siRNA | Sense (5’-3’) | GGAAGAGUGUCUGGAGCAATT |
|  | Anti-Sense (5’-3’) | UUGCUCCAGACACUCUUCCTT |
