# Supplementary material for: Transcriptional factor six2 promotes the competitive endogenous RNA network between CYP4Z1 and pseudogene CYP4Z2P responsible for maintaining the stemness of breast cancer cells
Source: J Hematol Oncol. 2019 Mar 4;12:23. doi: 10.1186/s13045-019-0697-6 (PMC6399913; doi:10.1186/s13045-019-0697-6)
Supplement: Supplementary file 5 — Table S5. Sequences of primers used for plasmid constructions. (DOC 40 kb) [file 13045_2019_697_MOESM5_ESM.doc]

**Additional file 5: Table S5.** **Sequences of primers used for plasmid constructions.**

| Name |  | Sequences |
| --- | --- | --- |
| PLVX-six2 | Forward (5’-3’) | CCGACTAGTATGTCCATGCTGCCCACCTTC |
|  | Reverse (5’-3’) | GCGGGATCCGGAGCCCAGGTCCACGAGGTT |
| PLVX-CYP4Z1-  3’UTR | Forward (5’-3’) | CCGCTCGAGGAGACAATTTTCCTACCAAAGGAAG |
|  | Reverse (5’-3’) | GCGGGATCCGCTTTATTCTCAGTTATCTTTCCCC |
| PLVX-CYP4Z2P-  3’UTR | Forward (5’-3’) | CCGCTCGAGCTTTCCAGATGGACGCTCCTTACCT |
|  | Reverse (5’-3’) | GCGGGATCCCCAGCAAGGAAATTAGAATTACTTAATCC |
| Plko.1six2 | Sense (5’-3’) | CCGGGCGAGCTCTACAAGATCCTCTCGAGAGGATCTTGTAGAGCTCGCTTTTTG |
|  | Anti-Sense (5’-3’) | AATTCAAAAAGCGAGCTCTACAAGATCCTCTCGAGAGGATCTTGTAGAGCTCGC |
| Plko.1CYP4Z1 | Sense (5’-3’) | CCGGCATTACCTTTCCAGATGGACTCGAGTCCATCTGGAAAGGTAATGTTTTTG |
|  | Anti-Sense (5’-3’) | AATTCAAAAACATTACCTTTCCAGATGGACTCGAGTCCATCTGGAAAGGTAATG |
| Plko.1CYP4Z2P | Sense (5’-3’) | CCGGCCACTCAGTATCTGCATTACTCGAGCCACTCAGTATCTGCATTATTTTTG |
|  | Anti-Sense (5’-3’) | AATTCAAAAACCACTCAGTATCTGCATTACTCGAGTAATGCAGATACTGAGTGG |
