# Supplementary material for: Transcriptional factor six2 promotes the competitive endogenous RNA network between CYP4Z1 and pseudogene CYP4Z2P responsible for maintaining the stemness of breast cancer cells
Source: J Hematol Oncol. 2019 Mar 4;12:23. doi: 10.1186/s13045-019-0697-6 (PMC6399913; doi:10.1186/s13045-019-0697-6)
Supplement: Supplementary file 6 — Table S6. Sequences of primers used for Luciferase reporter assay. (DOC 46 kb) [file 13045_2019_697_MOESM6_ESM.doc]

**Additional file 6: Table S6. Sequences of primers used for Luciferase reporter assay**

| Name |  | Sequences |
| --- | --- | --- |
| PGL3-CYP4Z1  -promoter | Forward  (5’-3’) | GGGGTACCCCTCACACCTCACAGAACTGGAGAAAGA |
|  | Reverse (5’-3’) | CCCTCGAGGGACCACCCCATCAGACAGGTTCACAT |
| PGL3-CYP4Z2P  -promoter | Forward  (5’-3’) | GGGGTACCCCGTGTCCCTTTTTCTCCACAGCCTCA |
|  | Reverse (5’-3’) | CCCTCGAGGGCAGCCTGTTCGTCAAATGCTCCTCA |
| PGL3-CYP4Z1  -promoter(1-500) | Forward  (5’-3’) | GGGGTACCTATTCCACAAGATAGAGAAAGAGAG |
|  | Reverse (5’-3’) | CCCTCGAGTATGTTGAATAGAAGTGGTGAAAGT |
| PGL3-CYP4Z1  -promoter(1-1000) | Forward  (5’-3’) | GGGGTACCTATTCCACAAGATAGAGAAAGAGAG |
|  | Reverse (5’-3’) | CCCTCGAGATCTAGTCATCCTTTGTCACACCTC |
| PGL3-CYP4Z1  -promoter(1-2000) | Forward  (5’-3’) | GGGGTACCTATTCCACAAGATAGAGAAAGAGAG |
|  | Reverse (5’-3’) | CCCTCGAGGTTAGTAACCACTGATGCCCTACCT |
| PGL3-CYP4Z2P  -promoter(1-500) | Forward  (5’-3’) | GGGGTACCTGAACTAATTTACATTCCTACCAACAG |
|  | Reverse (5’-3’) | CCCTCGAGCAATAACCTTCTCCATTTCAGCC |
| PGL3-CYP4Z2P  -promoter(1-1000) | Forward  (5’-3’) | GGGGTACCTGAACTAATTTACATTCCTACCAACAG |
|  | Reverse (5’-3’) | CCCTCGAGCGAAATGCTGCTGAAAGAAATC |
| PGL3-CYP4Z2P  -promoter(1-2000) | Forward  (5’-3’) | GGGGTACCTGAACTAATTTACATTCCTACCAACAG |
|  | Reverse (5’-3’) | CCCTCGAGCATTATGAATCCAGGAGTGAGTGAC |
| PGL3-CYP4Z2P  -promoter(1-2998) | Forward  (5’-3’) | GGGGTACCTGAACTAATTTACATTCCTACCAACAG |
|  | Reverse (5’-3’) | CCCTCGAGGGCAGCCTGTTCGTCAAATGCTCCTCA |
| PGL3-CYP4Z2P  -promoter(1200-2998) | Forward  (5’-3’) | GGGGTACCTGAACTAATTTACATTCCTACCAACAG |
|  | Reverse (5’-3’) | CCCTCGAGGGCAGCCTGTTCGTCAAATGCTCCTCA |
| PGL3-CYP4Z1  -promoter(MUT) | Forward  (5’-3’) | GGTCCTACACCGCCCTGAAACAGCAGCAGAGAC |
|  | Reverse (5’-3’) | GCGGTGTAGGACCCCAGAGGAGGAGATGGATGAT |
| PGL3-CYP4Z2P  -promoter(MUT  1125) | Forward  (5’-3’) | GGGGTACCTGAACTAATTTACATTCCTACCAACAG |
|  | Reverse (5’-3’) | CCCTCGAGCATTATGAATCCAGGAGTGAGTGAC |
| PGL3-CYP4Z2P  -promoter(MUT  1125) | Forward  (5’-3’) | CCACATCAGGAGCCAAATCAAGAAC |
|  | Reverse (5’-3’) | CTCCTGATGTGGATGTACTGGTATA |
| PGL3-CYP4Z2P  -promoter(MUT  2716) | Forward  (5’-3’) | GGGGTACCGTGGAAGCTGATGTGAGTTTTTTCAGGTGTTTCTGTGG |
|  | Reverse (5’-3’) | CCCTCGAGACTCACATCAGCTTCCACTGCGAAGGGAAATTA |
