# Supplementary figures and images for: Transcriptional factor six2 promotes the competitive endogenous RNA network between CYP4Z1 and pseudogene CYP4Z2P responsible for maintaining the stemness of breast cancer cells
Source: J Hematol Oncol. 2019 Mar 4;12:23. doi: 10.1186/s13045-019-0697-6 (PMC6399913; doi:10.1186/s13045-019-0697-6)

**A**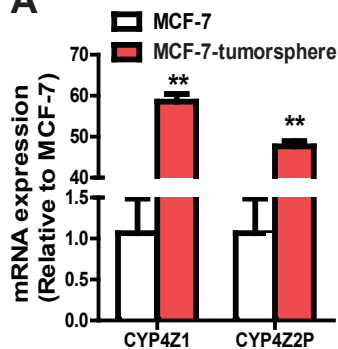**B**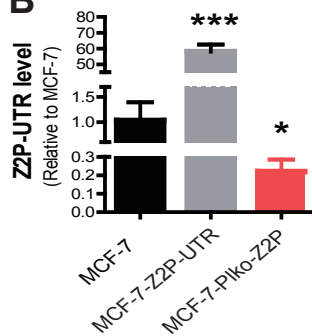**C**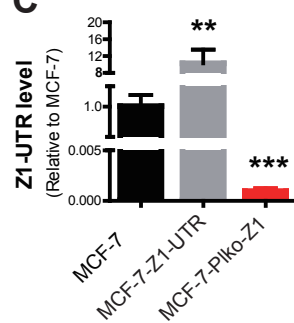**D**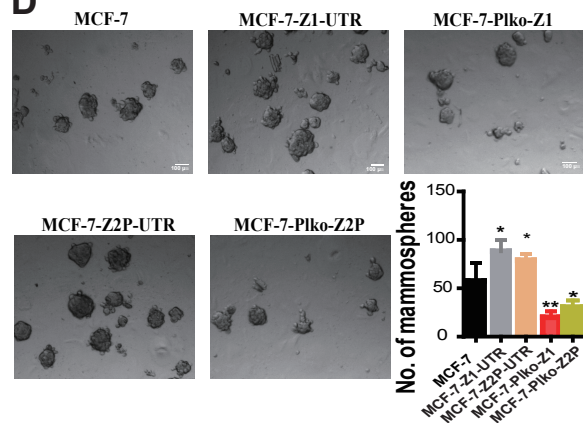**E**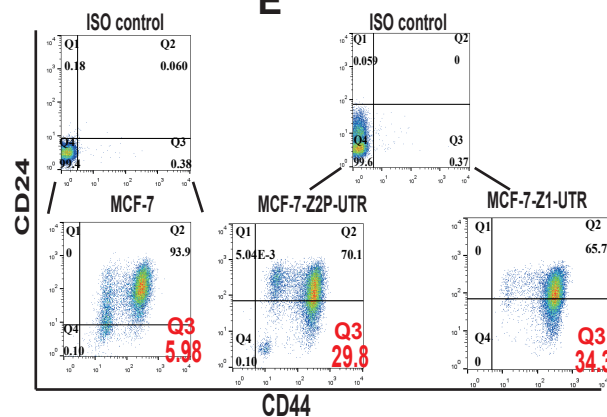**F**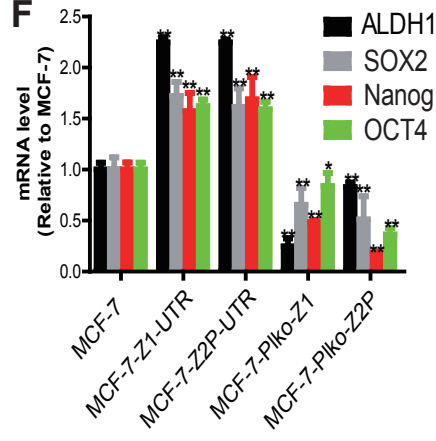**G**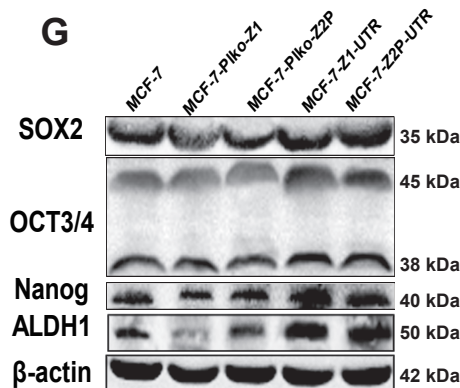

Supplement: Supplementary file 7 — Figure S1. CeRNET_CC promotes the stemness of MCF-7 cells in vitro. (A) The expression of CYP4Z2P and CYP4Z1 in MCF-7 and MCF-7-tumorsphere cells was detected by qRT-PCR. (B and C) The infection efficiency of MCF-7 cells with CYP4Z1- or CYP4Z2P-3′UTR stable overexpression (B) or knockdown (C) was detected by qRT-PCR. (D) Phase contrast images of mammospheres formed by stable expression cells depicted in B and C and quantification of spheres. (E) Representative FACS profile of cells described in B with CD24− and CD44+ markers. (F and G) The mRNA and protein expression of stemness markers (ALDH1, SOX2, OCT4 and Nanog) in cells described in B and C were examined by qRT-PCR and western blot analysis, respectively. The data are presented as the means ± SDs, n = 3, *P < 0.05, **P < 0.01, ***P < 0.001 vs. MCF-7. (PDF 5600 kb) [file 13045_2019_697_MOESM7_ESM.pdf]

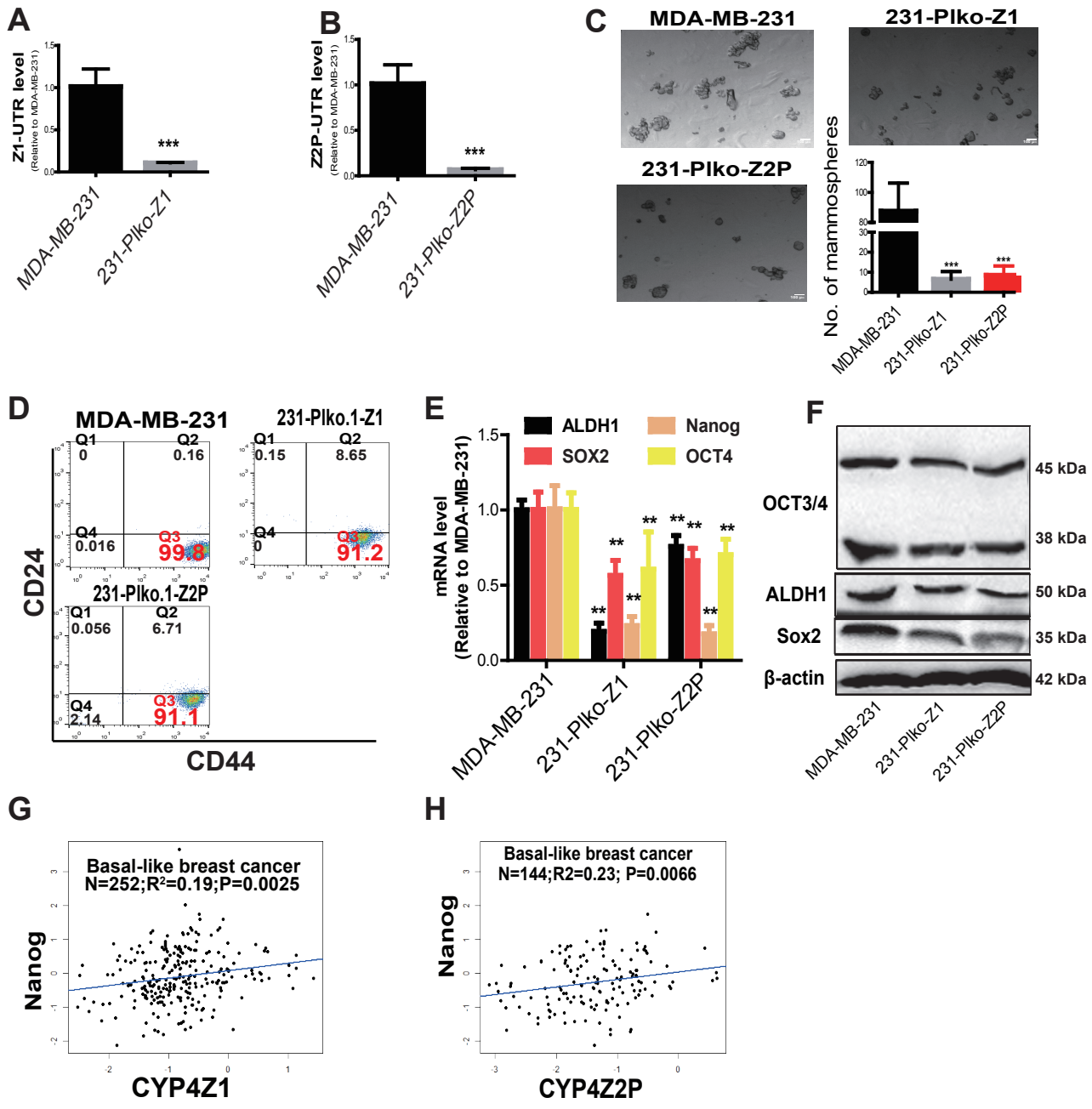

Supplement: Supplementary file 8 — Figure S2. CeRNET_CC promotes the stemness of MDA-MB-231 cells in vitro. (A and B) The infection efficiency of MDA-MB-231 cells with CYP4Z1- (A) or CYP4Z2P-3′UTR (B) stable knockdown was detected by qRT-PCR. (C) Phase contrast images of mammospheres formed by stable expression cells depicted in A and B and quantification of spheres. (D) Representative FACS profile of cells described in A and B with CD24− and CD44+ markers. (E and F) The mRNA and protein expression of stemness markers (ALDH1, SOX2, OCT4, and Nanog) in cells described in A and B. (G) Pearson correlation analysis of the expression of CYP4Z1 and Nanog in basal-like breast cancer (n = 252, P < 0.01). (H) Pearson correlation analysis of the expression of the pseudogene CYP4Z2P and Nanog in basal-like breast cancer (n = 144, P < 0.01). (PDF 3450 kb) [file 13045_2019_697_MOESM8_ESM.pdf]

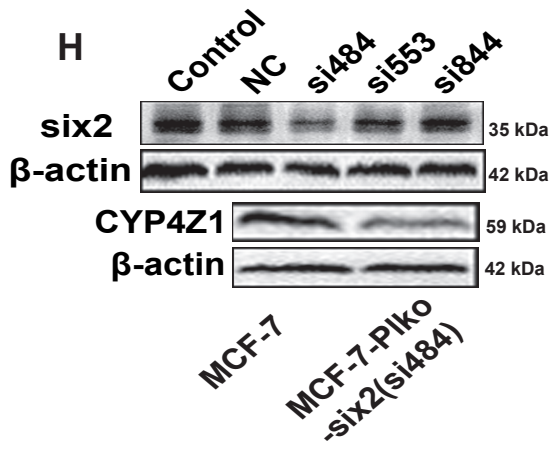

Supplement: Supplementary file 9 — Figure S3. Transcriptional factor six2 promotes the expression of CYP4Z1 and the pseudogene CYP4Z2P. (A) Computational analysis of the CYP4Z2P and CYP4Z1 promoters showed potential binding sites for six2. (B and C) Fragments of the CYP4Z2P and CYP4Z1 promoters were cloned into the luciferase reporter vector pGL3. MCF-7 cells were co-transfected with six2 and the luciferase constructs or the control construct. 72 h later, luciferase activity was measured. (D) Relative luciferase activity was detected in MCF-7 cells co-transfected with the six2 overexpression vector and CYP4Z1 and CYP4Z2P promoter vectors with six2 binding sites or mutated six2 binding sites. (E and F) The expression of CYP4Z2P, CYP4Z1, and six2 in MCF-7-six2 (E) and 231-six2 (F) cells was examined by qRT-PCR. (G and H) CYP4Z1 protein expression in MCF-7-six2 (G) and MCF7-Plko-six2 (H) cells was detected by western blot. The data were presented as the means ± SDs, n = 3, **P < 0.01 vs. control or MCF-7. (PDF 1270 kb) [file 13045_2019_697_MOESM9_ESM.pdf]

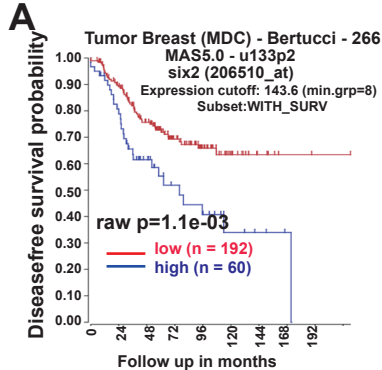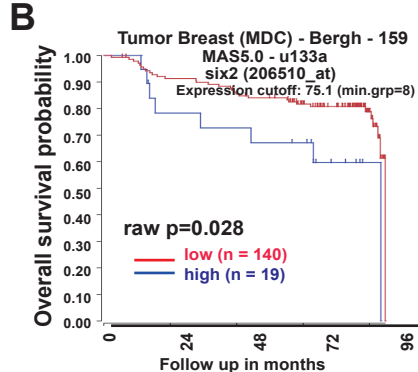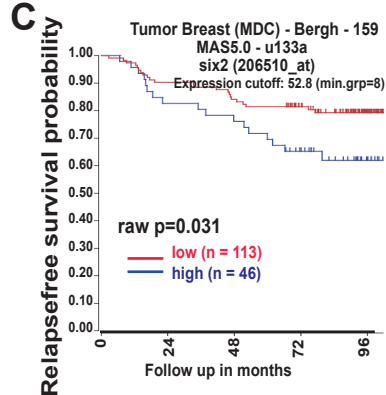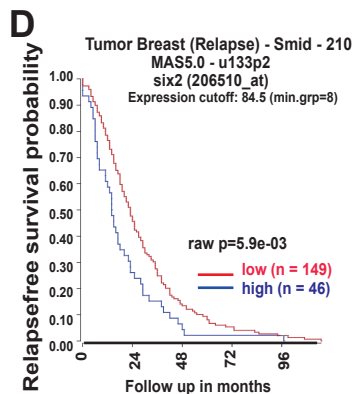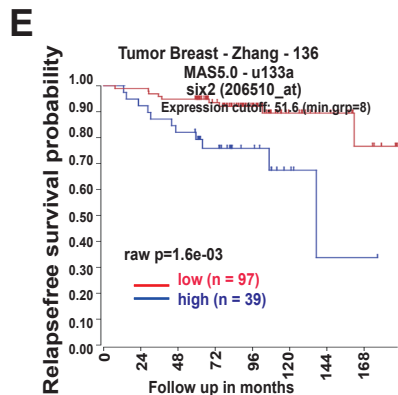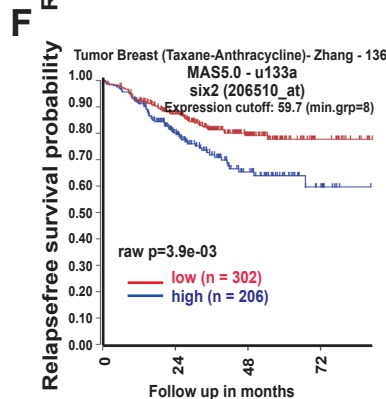

Supplement: Supplementary file 10 — Figure S4. The correlation between six2 expression and the survival of breast cancer patients. (A) KM-plotter survival curves showed the disease-free survival probability of patients separated into low and high six2 levels. (B) KM-plotter survival curves showed the OS survival probability of patients separated into low and high six2 level. (C and F) KM-plotter survival curves showed the RFS probability of patients separated into low and high six2 levels (http://hgserver1.amc.nl/cgi-bin/r2/main.cgi). (PDF 576 kb) [file 13045_2019_697_MOESM10_ESM.pdf]

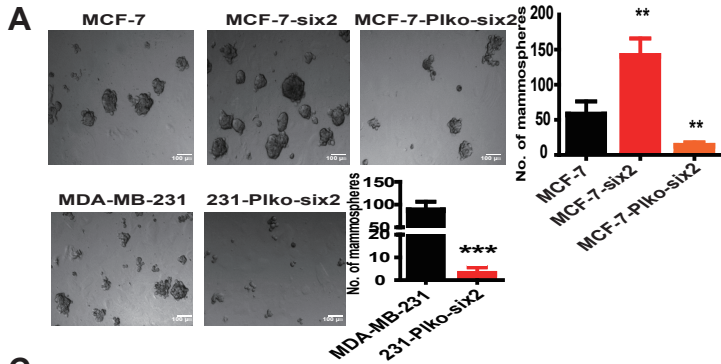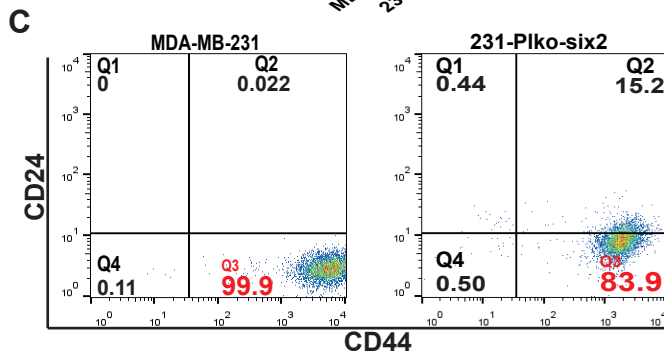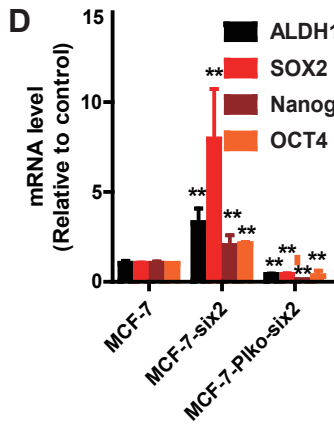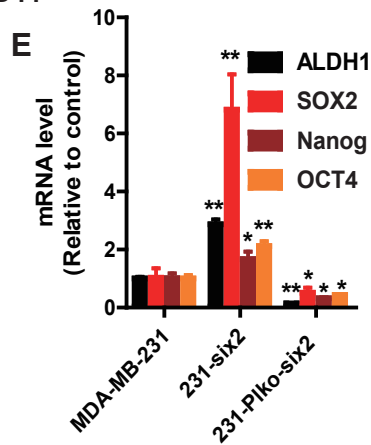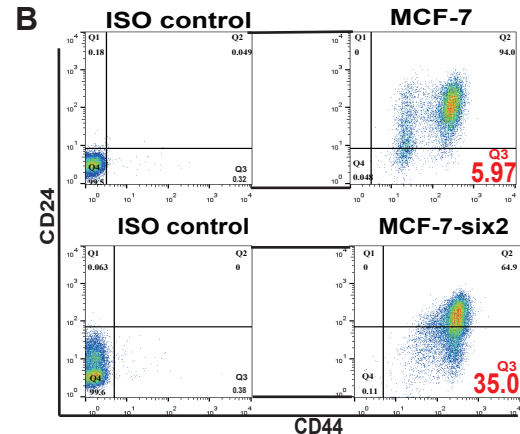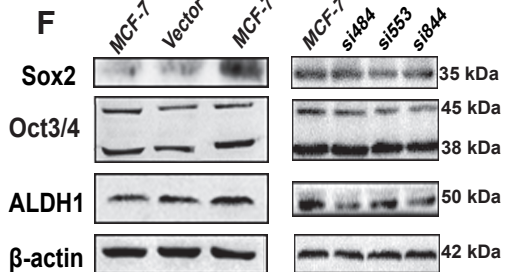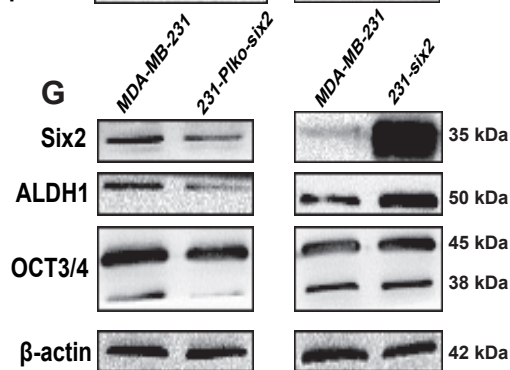

Supplement: Supplementary file 11 — Figure S5. Transcriptional factor six2 promotes the stemness of breast cancer cells in vitro. (A) Phase contrast images of mammospheres formed by MCF-7 cells with or without six2 overexpression or knockdown and MDA-MB-231 cells with or without six2 knockdown; spheres were quantified. (B and C) Representative FACS profile of MCF-7 cells with or without six2 overexpression (B) and MDA-MB-231 cells (C) with or without six2 knockdown, with the CD24− and CD44+ markers. (D and E) The mRNA expression of stemness markers (ALDH1, SOX2, OCT4, and Nanog) in MCF-7 (D) or MDA-MB-231(E) cells with six2 stable overexpression or knockdown was detected by qRT-PCR. (F and G) Cells depicted in D and E were subjected to western blot analysis and followed by detecting the expression of six2 and stemness markers (ALDH1, SOX2, and OCT3/4). The data are presented as the mean ± SD, n = 3, *P < 0.05, **P < 0.01, ***P < 0.001 vs. MCF-7 or MDA-MB-231. (PDF 4250 kb) [file 13045_2019_697_MOESM11_ESM.pdf]

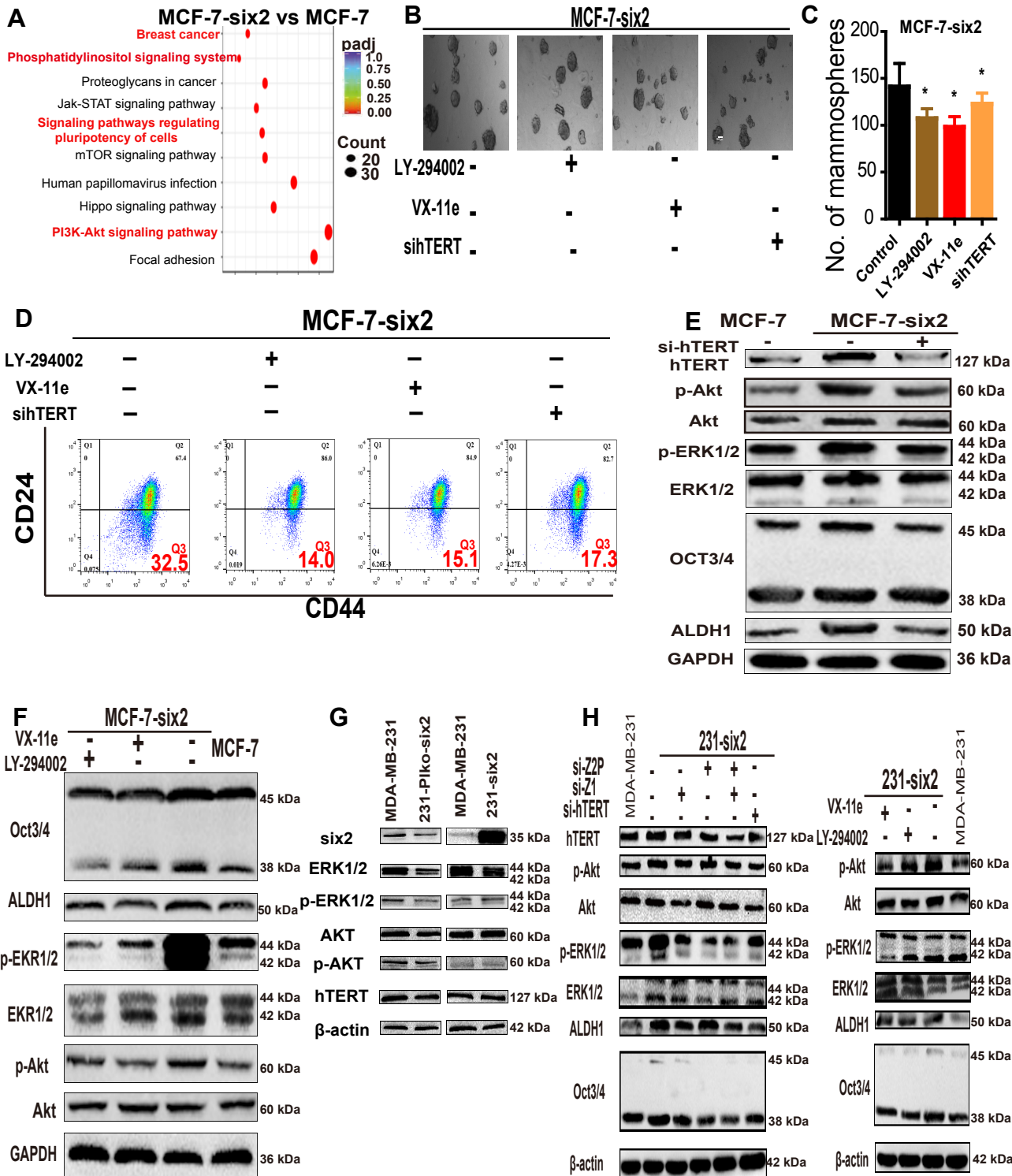

Supplement: Supplementary file 12 — Figure S6. Transcriptional factor six2 promotes the stemness of breast cancer cells partly through the hTERT/PI3K/Akt and ERK1/2 pathways. (A) Functional annotation analysis of genes coordinately activated by six2 overexpression. (B and C) Phase contrast images of mammospheres formed by MCF-7-six2 cells with LY-294002, VX-11e, or sihTERT treatment (B), and spheres were quantified (C). The data are presented as the means ± SDs, n = 3, *P < 0.05 vs. MCF-7-six2. (D) Representative FACS profile of cells described in B with CD24− and CD44+ markers. (E and F) Cells depicted in B were subjected to western blot analysis and followed by detecting the expression of p-Akt/p-ERK1/2 and stemness markers (ALDH1 and OCT3/4). (G) MDA-MB-231 cells with six2 stable overexpression or knockdown were subjected to western blot analysis and followed by detecting the expression of p-Akt/p-ERK1/2 and hTERT. (H) 231-six2 cells with LY-294002, VX-11e, or sihTERT, or si-Z1, or si-Z2P treatment were subjected to western blot analysis and followed by detecting the expression of p-Akt/p-ERK1/2 and stemness markers (ALDH1 and OCT3/4). (PDF 5040 kb) [file 13045_2019_697_MOESM12_ESM.pdf]

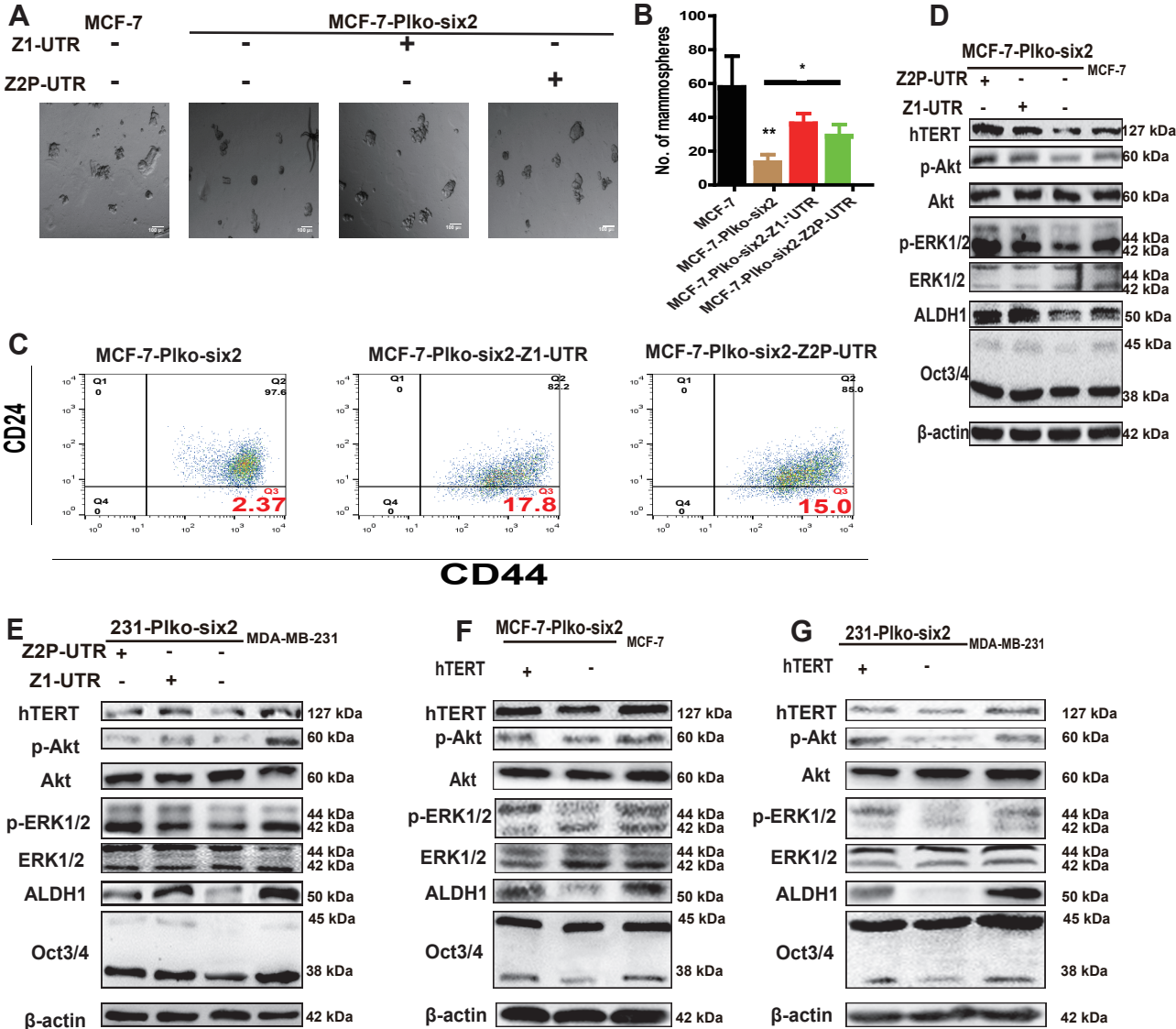

Supplement: Supplementary file 13 — Figure S7. CeRNET_CC is sufficient and necessary for six2-induced effects. (A and B) Phase contrast images of mammospheres formed by MCF-7-Plko-six2 cells with Z1-UTR or Z2P-UTR overexpression (A); spheres were quantified (B). The data are presented as the means ± SDs, n = 3, *P < 0.05, **P < 0.01 vs. MCF-7 or MCF-7-six2. (C) Representative FACS profile of cells described in A with CD24− and CD44+ markers. (D–G) MCF-7-Plko-six2 (D and F) and 231-Plko-six2 (E and G) cells with Z1-UTR, Z2P-UTR (D and E), or hTERT (F and G) overexpression were subjected to western blot analysis and followed by detecting the expression of p-Akt/p-ERK1/2 and stemness markers (ALDH1 and OCT3/4). The data were presented as the mean ± SD, n = 3, *P < 0.05, **P < 0.01 vs. MCF-7 or MCF-7-six2. (PDF 3760 kb) [file 13045_2019_697_MOESM13_ESM.pdf]

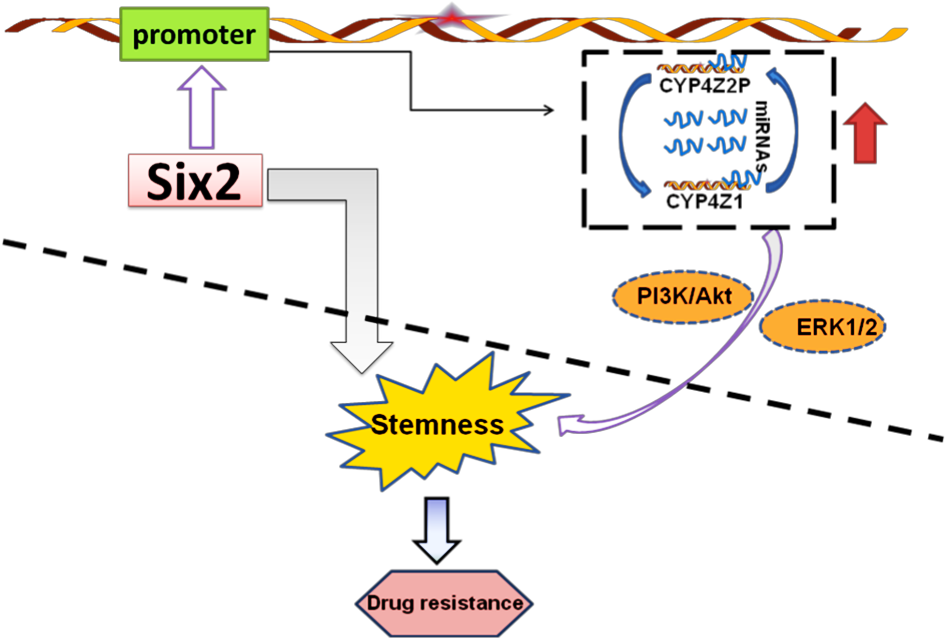

Supplement: Supplementary file 14 — Figure S8. Proposed model in which transcriptional factor six2-mediated regulation of ceRNET_CC is responsible for breast CSC formation and thus drug resistance. Transcriptional factor six2 induces the progression of ceRNET_CC by directly binding to the promoters of CYP4Z1 and the pseudogene CYP4Z2P. This six2/ceRNET_CC regulatory axis results in breast CSC progression and thus enhances drug sensitivity. (TIF 242 kb) [file 13045_2019_697_MOESM14_ESM.tif]
